# A half-site multimeric enzyme achieves its cooperativity without conformational changes

**DOI:** 10.1038/s41598-017-16421-2

**Published:** 2017-11-28

**Authors:** Mirella Vivoli, Jiayun Pang, Nicholas J. Harmer

**Affiliations:** 1 Department of Biosciences, University of Exeter, Stocker Road, Exeter, EX4 4QD UK; 2 Department of Pharmaceutical, Chemical and Environmental Sciences, Faculty of Engineering and Science,University of Greenwich, Medway Campus, Central Avenue, Chatham Maritime, Kent, ME4 4TB UK; 3 Living Systems Institute, University of Exeter, Stocker Road, Exeter, EX4 4QD UK

## Abstract

Cooperativity is a feature many multimeric proteins use to control activity. Here we show that the bacterial heptose isomerase GmhA displays homotropic positive and negative cooperativity among its four protomers. Most similar proteins achieve this through conformational changes: GmhA instead employs a delicate network of hydrogen bonds, and couples pairs of active sites controlled by a unique water channel. This network apparently raises the Lewis acidity of the catalytic zinc, thus increasing the activity at one active site at the cost of preventing substrate from adopting a reactive conformation at the paired negatively cooperative site – a “half-site” behavior. Our study establishes the principle that multimeric enzymes can exploit this cooperativity without conformational changes to maximize their catalytic power and control. More broadly, this subtlety by which enzymes regulate functions could be used to explore new inhibitor design strategies.

## Introduction

Cooperativity is a fundamental feature of many multi-subunit enzymes^1–3^. Cooperativity critically allows enzymes to make step-like rate responses to substrate concentration changes, and so offer rapid responses to changing cellular conditions. It is a key to link enzymes – the molecular building blocks of life – to the system-level events, such as signaling pathways and cellular responses. The effects of cooperativity have been characterized structurally in many systems^4^. Despite the simplicity of the underlying principle, different enzymes seem to adopt different strategies to accomplish cooperative conformational changes. Understanding how cooperativity is manifested in individual enzymes offers the opportunity to further understand the molecular basis of enzyme catalysis and facilitate drug discovery.

Both positive and negative homotropic cooperativity have been observed in a wide range of enzymes^5^. Positive cooperativity amplifies the sensitivity of the enzyme’s ligand binding capacity upon increase of ligand concentration and is important for maintaining the responsiveness of biological systems^6^. In negatively cooperative enzymes, binding of the first ligand reduces the affinity of binding of subsequent ligands, of particular relevance to ligand binding in signaling networks^7^.

Furthermore, it is not uncommon for proteins to display both positive and negative cooperativity to the same ligand. For example, yeast glyceraldehyde-3-phosphate dehydrogenase has long been studied as a model of cooperativity, as it has both positive and negative cooperativity for its substrate NAD^+^^8^. This was used as a partial proof for the validity of the “sequential” (Koshland-Némethy-Filmer) model of cooperativity^9^. Similarly, both positive and negative cooperativity has been observed in DNA binding proteins^10^. The extreme form of negative homotropic cooperativity is the so-called “half-site” reactivity, where enzyme active sites are paired, and only one can be active at a time^11,12^. Many enzymes consisting of tetramers or higher order multimers are made from two or more pairs of such coupled sites. These enzymes can then also display positive cooperativity towards the same ligand.

This half-site effect has generally been explained by an allosteric conformational change upon ligand binding that prevents binding at the paired site. Indeed, observation of half occupancy in a crystal structure is considered definitive of enzymes following the sequential model^11^. However, in some cases of half-site occupancy the conformational changes are too subtle for this definitive change to be observed. In these cases, molecular and quantum mechanics must be used to explain the half-site reactivity^13^.

The sugar isomerase (SIS) family of enzymes catalyze a wide range of isomerase reactions involved in sugar interconversions^14^. They generally bind to sugar phosphates, using the phosphate to provide affinity, and catalyzing reactions at other positions in the sugar^15,16^. This SIS family of enzymes show an interesting range of active site chemistries: the same evolutionarily conserved scaffold is used to coordinate different active site side chains to drive a variety of reactions throughout the family. This wide range of potential routes to control activity, and the differing numbers of subunits in different proteins (these enzymes are all either dimers or tetramers), offer evolution the opportunity to fine-tune the activity of SIS enzymes to cellular need.

One potentially interesting set of SIS enzymes are the heptose isomerases (GmhA)^14,16,17^. These tetrameric proteins catalyze the conversion of the pentose phosphate pathway intermediate sedoheptulose-7-phosphate (S7P) into d-*manno*-heptopyranose-7-phosphate (Supplementary Fig. 1). This product is then further modified to provide bacteria with heptoses for the production of polysaccharides. In particular, many Gram-negative bacteria incorporate heptoses into their lipopolysaccharide (LPS) core, and mutants with disrupted heptose production are highly susceptible to antibiotics^16^. Inhibitors of heptose biosynthesis could therefore potentiate other antibiotics. GmhA, a key enzyme along the heptose biosynthesis, has been identified as a novel target for antimicrobial intervention towards Gram-negative bacteria^18,19^.

Development of high affinity inhibitors against GmhA requires a firm understanding of its mechanism. The structure of the tetrameric GmhA has been solved from several organisms^20–24^. Among these, the structure with the most interesting features is GmhA from *B. pseudomallei*, a tetramer with a zinc ion at the heart of the active site, coordinating by the sidechains of H64, E68, Q175 and H183. All of these side chains are completely conserved amongst GmhA orthologues, and mutations in all of them significantly reduce activity. GmhA defines a distinct class of SIS domains in Pfam^25^. Unlike other SIS domains, which generally form dimers with active sites at considerable distances from one another (e.g.^26–28^), GmhA forms pairs of adjacent actives sites separated by 15 Å, linked by a solvent filled channel (Supplementary Fig. 2).

To further investigate GmhA’s catalytic mechanism and how it controls the activity of its tetrameric structure, we constructed and characterized GmhA and two variants with one or three active sites ablated. We observed clear evidence for positive cooperativity with a Hill coefficient of 1.5–2 in all samples. Surprisingly, we discovered that loss of one active site had no effect on activity; and that loss of three still left nearly 40% of wild-type activity. This strongly suggests that GmhA operates as a half-site reactivity enzyme. To test the molecular basis for this, we solved the structure of an inactive GmhA (Q175E) with substrate bound. This structure is almost indistinguishable from the previously solved unliganded, or product bound structures, precluding an allosteric conformational change that is often associated with enzyme cooperativity. Conversely, quantum mechanical (QM) calculations using active site models revealed that GmhA achieves negative cooperative control of its paired active sites through the solvent-filled channel that bridges these two active sites. Only one of such active site is able to bind to the substrate after energy optimization. Modeling of the global hydrogen bonding network suggested that positive cooperativity occurs between the two pairs of half-sites, with defined active sites showing cooperativity for each other. The half-site reactivity of GmhA gives it distinct advantage to control this step in heptose biosynthesis, hence the knowledge of cooperativity could be explored in inhibitor discovery.

## Results and Discussion

### GmhA shows both positive and negative cooperativity

**Figure 1:**
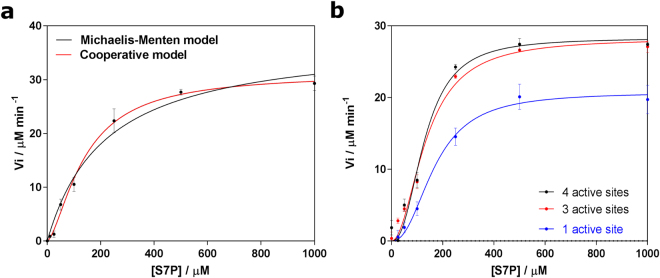
Cooperativity of GmhA. (**a**) Detailed assay of wild-type GmhA reveals that the enzyme shows positive cooperativity (>99% confidence in cooperative model, Akaike’s information criteria; n = 1.5 ± 0.2 (SEM)). (**b**) Comparison of GmhA with four active sites (black), three intact active sites (red) and one intact active site (blue; double concentration of enzyme added; cf. Supplementary Fig. 3) shows that each sample is cooperative (cf. Supplementary Fig. 4). Loss of one active site has no change from wild-type, within error; whilst over one third of wild-type activity remains after loss of three out of four active sites. All experiments were performed in triplicate. Error bars show standard error in the mean. Images generated using GraphPad.

Our previous study identified D61, which resides in the solvent filled channel, as an important residue in the activity of GmhA^20^ (Supplementary Fig. 2). The D61A mutation reduces the enzyme activity to 6% of wild-type – a greater effect than mutation of the zinc coordinating H64^20^. We therefore reasoned that there might be some cooperativity effects between the paired active sites in GmhA. We improved the enzyme assay in the present study, and were able to collect kinetic data sufficiently accurate to allow us to fit the substrate binding data to both Michaelis-Menten, and cooperative models (Fig. 1a). Statistical comparison of the fits indicated a 99% confidence in the cooperative model, with a Hill coefficient of 1.5 ± 0.2.

This Hill coefficient represents a significant level of cooperativity, and is not uncommon for enzymes. However, as GmhA has four subunits in all characterized orthologues, a greater level of cooperativity is generally possible. We reasoned that, as the SIS family of enzymes are generally either dimers or tetramers, cooperativity might only exist between the subunits that represent the minimal dimeric arrangement. In GmhA, these two active sites are linked by a water filled channel lined by D61. We therefore prepared GmhA chimeras with one or three active sites rendered inoperative by co-expression of D98N and E115K mutants: D98 assists in substrate binding and cyclization of the product (Supplementary Fig. 1b). The E115K mutation is a single nucleotide polymorphism found between the commonly used K96243 and 1710b strains of *B. pseudomallei*. E/K115 is located at the surface of the protein, well away from any active site, and the charge switch facilitates separation of chimeras (Supplementary Fig. 3). Analysis of the kinetics of each chimera again indicated that each enzyme fitted best to a cooperative model (Supplementary Fig. 4). This was the case even for the sample with a single intact active site. This suggests that the active sites that are not able to turn over the substrate might nevertheless bind substrate; and that this binding has the same cooperative effect as an intact active site.

Comparison of these chimeras to the wild-type enzyme showed that all three samples had a very similar *K*_*½*_ around 130 μM (Fig. 1b; Table 1). However, surprisingly, the sample with three active subunits showed a similar activity (*k*_*cat*_ 0.43 ± 0.02 s^−1^ per protomer; *k*_*cat*_ 0.57 ± 0.02 s^−1^ per intact active site) to the sample with all active sites intact (*k*_*cat*_ 0.44 ± 0.02 per promoter and active site; within error of the wild-type sample); and the sample with only one active subunit showed approximately one-third of the wild-type activity (*k*_*cat*_ 0.144 ± 0.008 s^−1^ per promoter; *k*_*cat*_ 0.57 ± 0.03 s^−1^ per intact active site). A drop in activity would be expected on the loss of one active site, especially in the light of the positive cooperativity observed, as indicated by a Hill coefficient greater than 1. These results suggested that, as well as the positive cooperativity observed, there might be negative cooperativity occurring between pairs of active sites, so that loss of one active site would not affect the rate.

**Table 1:** Kinetic parameters of GmhA variants.

	*k*_*cat*_ (s^−1^) per protomer	*k*_*cat*_ (s^−1^) per active site	*K*_*1/2*_ (µM)	*h*	Model probability	
					Michaelis-Menten	Cooperative
WT^a^	0.5 ± 0.1	0.5 ± 0.1	500 ± 200	1^b^	ND	ND
D61A^a^	0.02 ± 0.01	0.02 ± 0.01	300 ± 500	1^b^	ND	ND
WT^a^	0.48 ± 0.02	0.48 ± 0.02	140 ± 20	1.5 ± 0.2	1.8%	98.2%
Hybrid (4 active sites)^c^	0.44 ± 0.01	0.44 ± 0.01	130 ± 10	2.4 ± 0.3	<0.01%	>99.99%
Hybrid (3 active sites)	0.43 ± 0.01	0.57 ± 0.02	140 ± 10	2.0 ± 0.2	<0.01%	>99.99%
Hybrid (1 active site)	0.148 ± 0.008	0.57 ± 0.03	170 ± 20	2.3 ± 0.4	0.13%	99.87%

Model probabilities were calculated using the Akaike information criterion in Graphpad. Errors shown are SEM.

^a^Data taken from^20^.

^b^ *h* was assumed to be 1 in previously published data.

^c^i.e. an E115K mutant homotetramer.

### Structures of B. pseudomallei GmhA active site mutants provide insight into the mechanism of cooperativity

**Figure 2:**
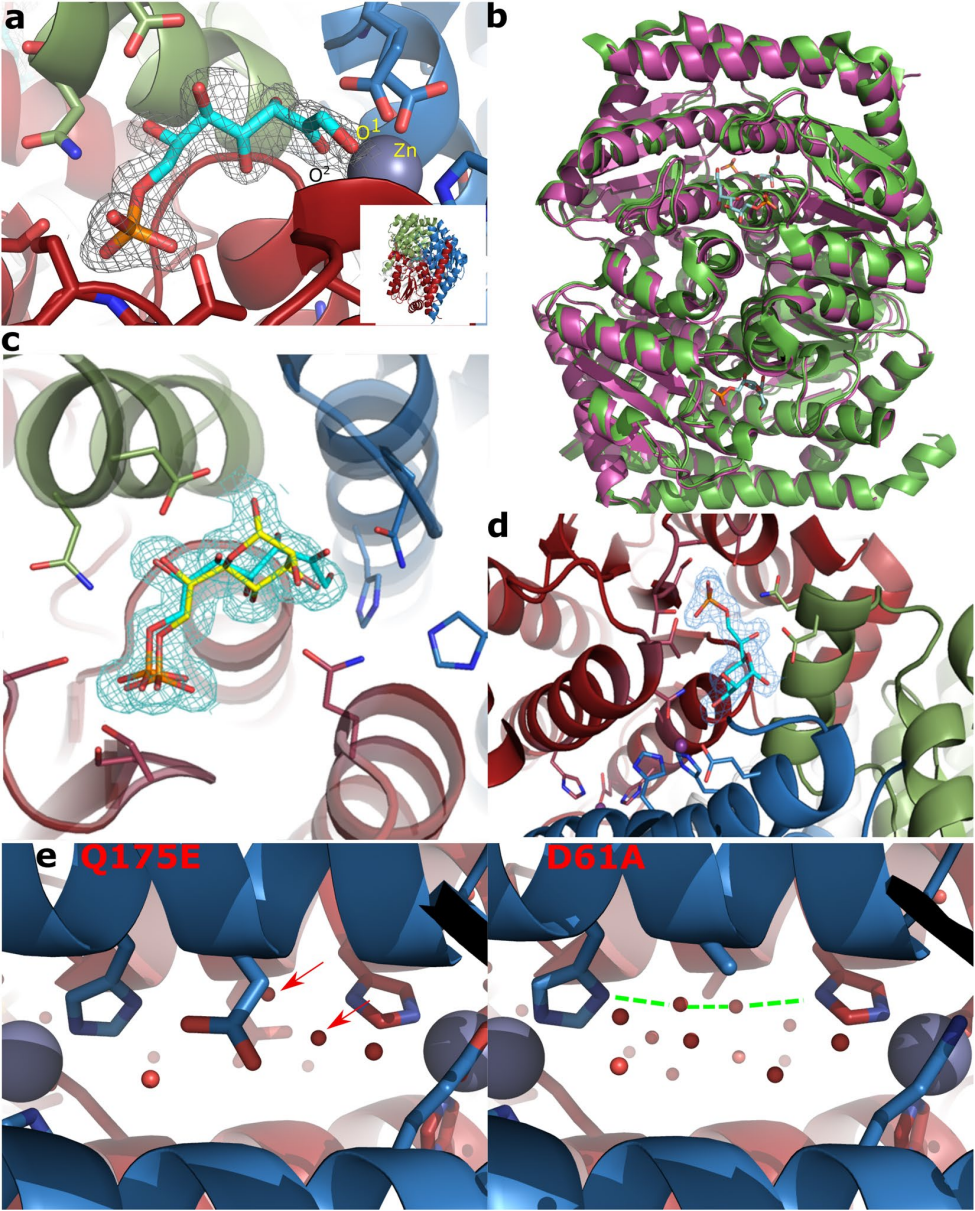
Structures of GmhA mutants soaked with substrate reveal substrate binding modes. (**a**) The structure of the GmhA Q175E mutant soaked with S7P shows substrate in a “pre-reaction” conformation, poised for reaction. The O^1^ and O^2^ atoms of S7P make coordinate bonds with the zinc ion, which adopts an octahedral conformation. Inset: GmhA overall structure in the same orientation as images A-C. (**b**) The superimposed structures of apo (green; PDB ID: 2X3Y) and substrate bound (PDB ID: 5LTZ, magenta, this study) GmhA. There is no significant alteration in the structure, with an RMS deviation of 0.266 Å between the 4,659 aligned atoms in the structures. (**c**) The structure of the GmhA E68Q mutant soaked with S7P shows a mixture of substrate in a non-reactive configuration (cyan), and product (yellow; in the conformation previously observed^20,22^). (**d**) The structure of the GmhA D61A soaked with S7P shows product, in the same conformation as the product seen in the E68Q mutant (**c**). (**e)** Comparison of the channel between pairs of active sites in the Q175E (left) and D61A mutant (right) shows a significant alteration in the water structure in the mutant. Colors: GmhA chain A, sky blue; chain B, white; chain C, green; chain D, brick red; substrate S7P carbon, cyan; product M7P carbon, yellow; oxygen, red; nitrogen, blue; phosphorus, orange; zinc, purple sphere; water, red sphere. Density shown as blue netting is the 2*F*_*o*_ *-F*_*c*_ map, contoured at 1 σ. Figure generated using PyMOL.

Two previous structures of GmhA have only shown the product bound to the active site^20,22^. A substrate bound structure was obtained, albeit in an “open”, inactive conformation^22^. To assist in modeling the catalytic complex of GmhA, we determined in the present study the crystal structure of four active site mutants shown to affect activity (D61A, H64Q, E68Q, and Q175E), soaked with the reaction substrate S7P (Supplementary Table 1). Pleasingly, one of these mutants (Q175E) showed the substrate clearly in all four active sites, in a conformation suggestive of a pre-catalysis complex (Fig. 2a). The two substrate oxygen atoms involved in the reaction are both forming coordination bonds to the zinc ion at the heart of the active site of *B. pseudomallei* GmhA, at distances between 2.3–2.6 Å. The zinc ion coordination changes to octahedral from the pentahedral and tetrahedral coordination previously observed^20^ to accommodate these additional ligands. Comparison of the substrate bound structure to either unliganded or product bound structure showed negligible differences in the overall conformation of the enzyme (RMS deviation of 0.266 Å; Fig. 2b; Supplementary Fig. 5). As conformational changes that break symmetry are considered diagnostic of half-site enzymes, this suggested that a different, novel mechanism must be causing the half-site effect in GmhA.

Surprisingly, the structure of the E68Q (Fig. 2c) mutant showed a mixture of substrate and product, with some two active sites showing approximately equal quantities of both and the other two showing an excess of product. The substrate was not interacting with the zinc, but was located out of coordination distance from this, with the two reactive oxygen atoms O^1^ and O^2^ displaced by an average of 3.2 Å and 4.5 Å respectively. Similar results were seen in all four active sites in the crystal structure. In contrast, the structure of H64Q showed the substrate in a similar location to that seen in the E68Q mutant (Supplementary Fig. 6), whilst the structure of the D61A mutant showed strong density for the product (Fig. 2d). Inspection of the solvent filled channel in the D61A mutant showed that the channel water structure was significantly disrupted (Fig. 2e), shifting from an asymmetric water distribution to a symmetric distribution. As this was the only apparent difference between this structure and the wild-type complex, this suggested that this channel might be of functional significance for GmhA. Taken together with our earlier results on the cooperativity of GmhA (Fig. 1, Table 1) and the activity of D61A and H64Q mutants (6% and 14% of wild-type respectively^20^), we reasoned that GmhA may use the short-range hydrogen bonding network between the water molecules in this channel, D61 and H64 (which coordinates to the zinc in the active site) to enable communication between the two paired active sites. This may lead to either the positive or negative cooperativity.

### Quantum mechanical calculations of the GmhA active site models

**Figure 3:**
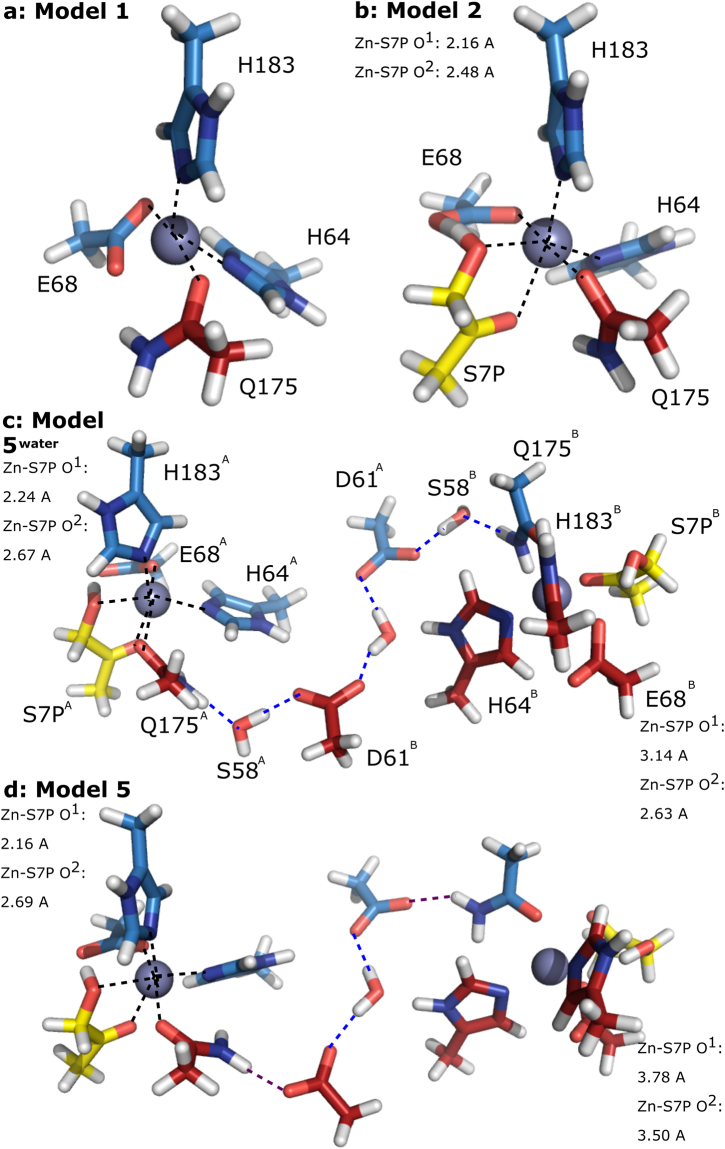
Quantum mechanical modeling of the GmhA active site. (**a**) In the absence of substrate, the zinc ion in the GmhA active site models to a tetrahedral configuration (black dashed lines showing coordination bonds), reflecting crystal structures. (**b**) On adding substrate (modelled as the final three carbons of S7P), the zinc coordination changes to an octahedral configuration, reflecting the configuration seen in the Q175E mutant (Fig. 2a). (**c**) When a pair of active sites is modeled with the connecting water channel, including water atoms as a proxy for S58, one zinc ion (left) adopts an octahedral configuration, whilst the other (right) adopts a tetrahedral coordination, pushing the substrate S7P away from the zinc ion. The hydrogen bonding network linking the two active sites is shown through blue dashed lines. (**d**) A similar model to (**c**) omitting S58, shows a similar pattern of zinc coordination and hydrogen bonding in the channel linking the active sites. However, in this model the nitrogen atom of Q175 forms a hydrogen bond with D61 (unlike in the crystal structure; purple dashes), rather than adopting a position to act as a hydrogen bond donor to the substrate and intermediate. Colors: chain 1 carbon, sky blue; chain 2 carbon, brick red; substrate carbon, yellow; oxygen, red; nitrogen, blue; hydrogen, white; zinc, gray sphere.

Armed with the structure of the likely substrate posture before catalysis based on our mutant crystal structures, we performed QM geometry optimizations of the active site models. The purpose of these models is to provide further insights into how the solvent filled channel, in particular H64, D61 and the water molecules, exerts influence on the coordination of zinc, and hence the substrate binding. Initial studies on the single active site model (Model 1, Fig. 3a) were in agreement with the crystal structures: in the absence of substrate, a tetrahedral geometry is preferred in the coordination state of H64, E68, Q175 and H183 to the active site zinc ion. This changes to octahedral in the presence of substrate S7P, with O^1^ and O^2^ of the substrate making coordination bonds with zinc (Zn-O^1^ distance = 2.16 Å and Zn-O^2^ distance = 2.48 Å; Model 2; Fig. 3b). Following this, we built a model of paired active sites (Model 3), linked by a water molecule and D61 from the solvent channel (Supplementary Fig. 2). In the absence of substrate, the two active sites are largely equivalent. When one substrate binds to the paired model (Model 4; Supplementary Table 2), the zinc ion in one active site changes to octahedral coordination accommodating two substrate oxygens as ligands; the difference between the two paired active sites becomes apparent when a second substrate is added to the model (Model 5 and Model 5^*water*^): here, the Zn-O^1^ distance is significantly increased from ~2.16 Å as seen from Model 2 and 2.19 Å in Model 4 to 3.14 Å in Model 5^*water*^. The difference is even more pronounced in Model 5, in which the water molecule bridging between Q175 and D61 in Model 5^*water*^ is removed and the sidechain of Q175 forms hydrogen bond directly to D61. There is a strong preference not to bind to substrate at the second active site (Fig. 3d, Supplementary Table 2). Substrate in the second active site is pushed away from the zinc (Zn-O^1^ distance = 3.78 Å and Zn-O^2^ distance = 3.50 Å), in a manner reminiscent of the substrate observed in the structure of the E68Q mutant (Fig. 2c).

Zinc’s charge is related to its electron-accepting ability as a Lewis-type catalyst in metalloenzymes. Interestingly, both zinc ions within the unliganded paired active site models exhibit slightly increased charges (1.292 and 1.302 in Model 3) compared to their counterpart in the unliganded single active site model (1.271 in Model 1; Table 2). In addition, the zinc ions in Model 3 also have smaller condensed Fukui indices *f* ^+^_Zn_ (0.344 and 0.307, respectively) than Model 1 (0.498), indicating that the paired active site environment is better at attuning Zn’s Lewis acidity by maintaining its net charge. It is conceivable that the more positively charged zinc in the paired active sites would make the intermediate (probably a negatively charged ene-ol) more favorable, and so may increase the rate of the reaction. On the other hand, loss of the coupling between the paired active site by means of the D61A mutation leads to an enzyme that is still active, and has no half-site effect, but is less active overall (*i.e*. D61A mutant reduces enzyme activity to 6% of wild-type).

**Table 2:** Zinc charges and condensed Fukui indices in the quantum mechanical models.

M05-2X/6-31 + G*	*q*_Zn_(n)	*q*_Zn_(n + 1)	*|f* ^+^_Zn_|
Model 1	1.271	0.773	0.498
Model 3 ^*active site A*^	1.292	0.948	0.344
Model 3 ^*active site B*^	1.302	0.995	0.307

The QM calculation of the paired active sites strongly support a model where D61, linking by a network of hydrogen bonds involving water molecules in the solvent channel and active sites, the active site H64 and Q175, tunes the behavior of the zinc ions, such that only one zinc can support a pentahedral or octahedral geometry. Consequently, the enzyme elegantly restricts activity to one of these two active sites at a time. These observations explain the negative cooperativity observed in the chimeric mutants (Fig. 1b). The QM calculations are consistent with the significantly reduced rate in the E68Q mutant, which showed a mixture of product and substrate in the active site. This mutant appears to have engaged one substrate productively, and one non-productively, in each pair of active sites. In the crystal, these are averaged over all of the available enzymes.

### Global hydrogen bond analysis indicates a preference for binding substrate molecules on opposite sides of the GmhA tetramer

**Figure 4:**
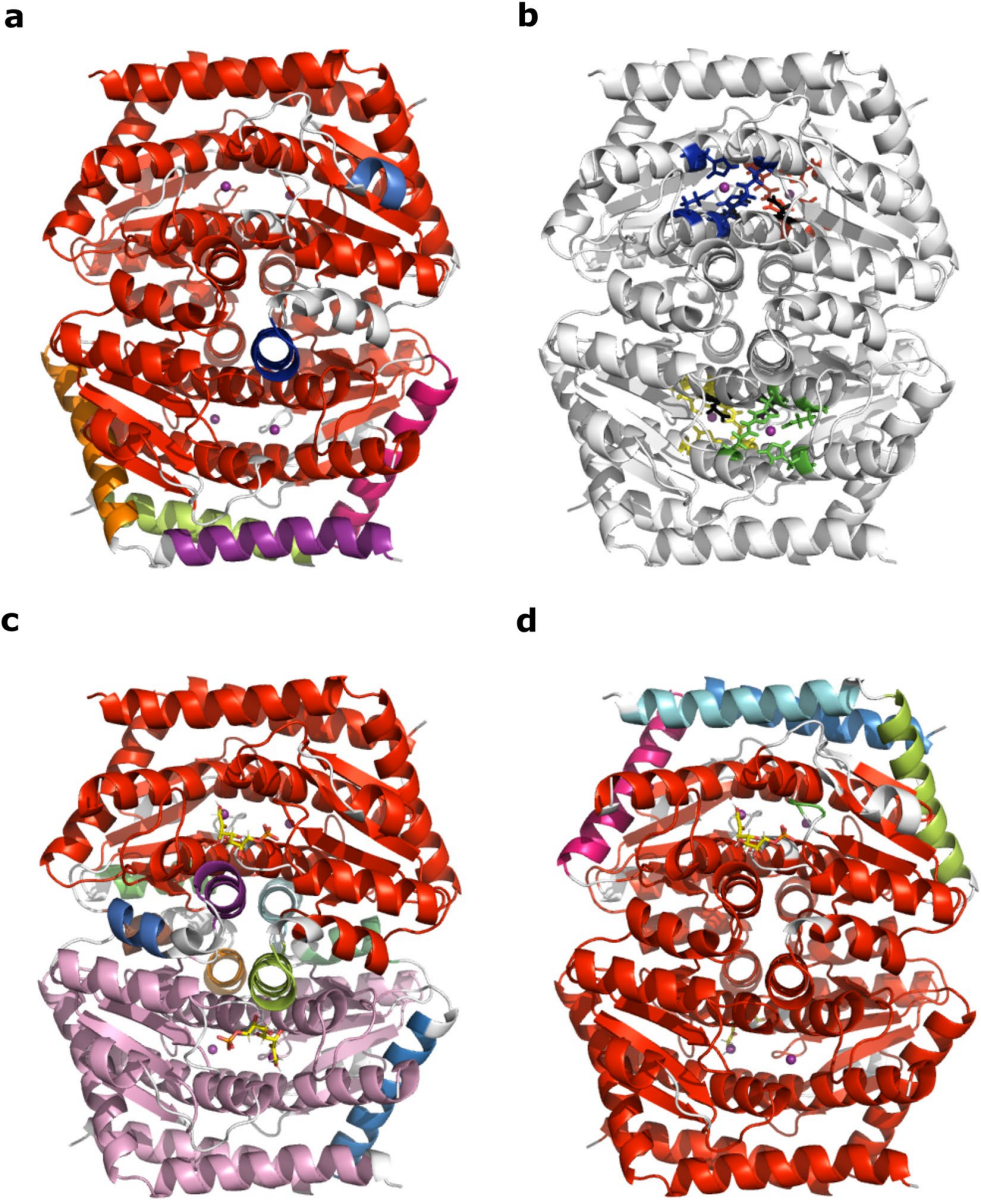
Modeling of the global hydrogen bond network of GmhA. The global hydrogen bonding network was determined using Proflex^29^. Images show the distribution of the protein structure into units that form a rigid hydrogen bonding network at 1.8 kcal/mol (just before the main network of unliganded GmhA loses integrity), in a typical case for each ligand state. Each independent unit is indicated by a different color, with the major unit shown in red. Colors correspond to the detailed data shown in Supplementary Fig. 7. (**a**) Global hydrogen bonding network of unliganded GmhA. (**b**) Schematic of the GmhA active sites. The active sites shown by the yellow/green and red/blue active sites are paired to one another through a water filled channel (cf. Fig. 2e). The green/blue and yellow/red active sites are located on the same face of the GmhA tetramer; ligand binding at both of these sites leads to a loss of structural stability (**c**) where the GmhA hydrogen bonding network loses integrity at an energy 0.25 kcal/mol lower than wild-type. The green/red and yellow/blue active sites are unpaired, and located on opposite faces of the tetramer; ligand binding at both of these sites (**d**) leads to an increase in stability of the major hydrogen bonding network of 0.2 kcal/mol compared to wild-type.

The QM calculations clearly explain the basis for negative cooperativity in GmhA. However, they cannot explain the positive cooperativity observed. The two pairs of active sites with positive cooperative effect are too far apart to construct a QM model of both pairs. Therefore, we instead considered the global hydrogen bonding network of the enzyme upon substrate binding, expecting that cooperative binding events would alter this network. The global hydrogen bonding network of GmhA is surprisingly delicate. The main hydrogen bonding network (Fig. 4a, red)^29^ loses integrity and breaks into a series of smaller rigid elements when hydrogen bonds that are only 1.87 ± 0.04 kcal/mol or weaker are broken (average of ten simulations; cf. WcbL, the next enzyme in the pathway, where the main network is stable until the loss of hydrogen bonds 4.1 kcal/mol or weaker^30^; Table 3; Fig. 4; Supplementary Fig. 7). The GmhA network shows a trend to lose stability when one substrate binds to GmhA (~0.1 kcal/mol; not significant [ANOVA test between all pairs of structures tested]). When a second substrate binds on the same face of the GmhA tetramer (Fig. 4b), there is a further small loss in the stability of the network (~0.1 kcal/mol; *P* < 0.01 compared to unliganded), consistent with negative cooperativity. In contrast, when the second substrate molecule binds on the opposite face of the GmhA tetramer (Fig. 4c), there is a clear gain in the stability of the hydrogen bonding network (~0.3 kcal/mol; *P* < 0.001 compared to the single substrate bound), as expected for positive cooperativity. Figure 4 and Supplementary Fig. 7 also illustrate that GmhA is dominated by a single integrated hydrogen bonding cluster with limited relative motions (shown in red in Fig. 4 and Supplementary Fig. 7), which stays intact upon substrate binding at different active sites. Both positive and negative cooperativities of GmhA are associated with truly global changes in the hydrogen bonding network, which involves very small increase and decrease of many hydrogen bonds’ energies.

**Table 3:** Strength of GmhA global hydrogen bond networks upon ligand binding.

Ligand	Minimum hydrogen bond energy (kcal/mol)	Significance of differences (ANOVA)
1: None	1.87 ± 0.04 (1.80–1.94)	n/a
2: One molecule of S7P	1.74 ± 0.03 (1.67–1.81)	1: n.s.
3: Two molecules of S7P (*cis*)	1.62 ± 0.02 (1.59–1.65)	1: ** 2: n.s.
4: Two molecules of S7P (*trans*)	2.08 ± 0.08 (1.92–2.24)	1: * 2: *** 3: ****

The minimum hydrogen bond energy at which GmhA forms a single rigid, integrated structural network was determined before and after adding substrate. A first S7P molecule was arbitrarily placed in the active site at the interface between chains A, C and D (blue active site in Fig. 4b). A second molecule was placed in the active site at the interface between chains B, C and D (green active site in Fig. 4b) to create a “*cis*” complex with the S7P molecules on the same face of GmhA; or in the active site at the interface between chains A, B and C (yellow active site in Fig. 4b) to create a “*trans*” complex with S7P molecules on opposite faces of GmhA. Values given are the mean from ten technical replicates calculated using HETHER^41^, with the standard error in the mean. 95% confidence intervals are given in parentheses. The significance of differences between the four groups was determined using ANOVA (implemented in Graphpad v7.0.3). n.s: not significant; *P < 0.05; **P < 0.01; ***P < 0.001; ****P < 0.0001.

These data are consistent with both the positive cooperativity and negative cooperativity demonstrated by the quantum mechanical calculations: they suggest a model in which binding of substrate at one active site deters binding at either of the neighboring active sites, but promotes binding at the non-neighboring active site. The coupled paired active site is deterred by the zinc ion in this active site strongly preferring the tetrahedral coordination; whilst the other neighboring active site is deterred by the unfavorable effects on the global hydrogen bonding network. In contrast, both of these effects will favor binding at the non-neighboring active site, as the global hydrogen bond network shows a substantial improvement (releasing energy for binding), and the coupling of the active sites will favor binding of a substrate molecule to the zinc ready for catalysis. As GmhA is a slow enzyme (with a turnover number of less than 1 s^−1^), there is likely to be significant binding and release of substrate before a catalytic event takes place. The H-bond modeling observations therefore strongly support the biochemical observation of positive cooperativity with a Hill coefficient of 1.5–2, supporting a robust cooperativity between non-neighboring active sites. The data therefore suggest a model whereby alternating pairs of active sites on opposite sides of the molecule will engage with substrate and form a productive enzyme-substrate complex. The other pairs of active sites will not productively engage substrate, but instead provide catalytic power through the coupling of the active sites that exceeds the loss of activity from the non-use of these active sites. This enzyme therefore employs an elegant mixture of positive and negative cooperativity to tune its catalysis using subtle alterations to its hydrogen bonding network.

### Conclusions and future prospects

Here, we show that GmhA is a half-site enzyme with positive homotropic cooperativity. The enzyme achieves this through an intricate network of hydrogen bonds and coupling of the active sites controlled by a unique water channel, exploiting the three dimensional structure of the protein to maximize its catalytic power and control. Our studies of GmhA provide a new mechanistic framework for positive and negative cooperativity, which may be available to many multimeric enzymes. We expect that further biochemical and modelling studies will reveal that these effects have evolved in many enzyme families. More generally, it is increasingly recognized that understanding subtle dynamical changes of protein structures can be the key to design powerful inhibitors. The subtleties by which GmhA regulates its substrate binding could be used to guide the design of highly selective inhibitors for multimeric enzymes.

## Online methods

### Preparation of GmhA, WcbL and WcbN

Full length sedoheptulose-7-phosphate isomerase (GmhA; Fig. 1), d,d-heptose-7-phosphate 1-kinase (WcbL) and d,d-heptose-1,7-bisphosphate 7-phosphatase (WcbN) from *B. pseudomallei* strain K96423 (genomic DNA a gift of R. Titball, University of Exeter) were prepared as previously described^20^. Mutants of GmhA were prepared using the QuikChange Lightning kit (Agilent), and confirmed by sequencing. Hybrids of E115K and D98N were prepared by subcloning the D98N mutant into the pCDF-Duet vector (Novagen), using the *Nco*I and *Hind*III sites (*Nco*I covers the start codon of GmhA in the cloned protein; the *Hind*III site is provided by pNIC28-Bsa4, after the stop codon). pNIC28-GmhA E115K and pCDF-Duet-GmhA D98N were transformed into Rosetta (DE3) cells (Merck), and grown in ZYM-5052 media^31^ supplemented with 100 μg/mL kanamycin, 25 μg/mL spectinomycin, and 10 μg/mL chloramphenicol. Cells were grown, harvested, lysed and initially purified as described for wild-type GmhA^20^. For preparation of hybrid GmhA, the size exclusion column was eluted isocratically with 10 mM Tris pH 8.0, 50 mM NaCl (buffer A). The hybrid proteins were separated using a 1 mL HiTrap Q HP column. The column was equilibrated in buffer A, and the protein eluted using a gradient to 288 mM NaCl over 50 mL. Pooled fractions were dialysed twice against a 50-fold excess of 10 mM Hepes pH 7.0, 0.5 M NaCl (buffer B). GmhA for enzyme assays was concentrated as necessary using a Vivaspin centrifugal concentrator (Vivascience), and stored at −20 *°*C with 20% (v/v) glycerol added.

### Structure Determination of GmhA

All crystals were grown using the microbatch method, and were prepared using an Oryx6 crystallization robot (Douglas Instruments). Tag-removed GmhA mutants at 10 mg/mL were mixed with an equal volume of crystallization solutions, and crystals were grown at 4 °C. Prior to flash freezing, crystals were soaked in cryoprotectant solutions containing S7P (Supplementary Table 1). X-ray diffraction data sets were collected at 100 K at beamlines I02 and I03 of the Diamond synchrotron. Single wavelength datasets were collected for all crystals (Supplementary Table 1). To determine the identity of the binding metal, an EXAFS scan was performed. An energy of 20 keV was used for the EXAFS scan. Data were processed using Xia2 version 0.3.8^32^, XDS^33^ and Aimless^34^. Model building and refinement of the structures was performed using Coot version 0.8^35^, Refmac version 5.8 and PHENIX version 1.9^36^. Structures were validated using PHENIX, Coot, Rampage^37^ and MolProbity^38^. Structural images were prepared using the PyMOL molecular graphics system (DeLano Scientific).

### GmhA Enzyme Activity Assay

GmhA activity was monitored by coupling product formation to WcbL and WcbN and monitoring P_i_ release, in an analogous manner to the methods of DeLeon *et al*.^17^. Briefly, a reaction mixture was formed consisting of 20 mM HEPES, pH 8.0, 10 mM MgCl_2_, 10 mM KCl, 30 μg/mL WcbL, 40 μg/mL WcbN, and 25–50 μg/mL GmhA in a total volume of 50 μL. Reactions were initiated with 50 μL of ATP at 1 mM final concentration and S7P (Carbosynth) for final concentrations ranging from 0 to 1 mM (all concentrations given in the final 100 μL mixture). Samples were incubated at 37 °C for 10 min, and the reaction was terminated by heating to 70 °C for 5 minutes. The phosphate concentration was determined by the addition of 17 μL of Pi Colorlock Gold (Innova Biosciences) to 68 μL of reaction product. After 5 minutes, 6.8 μL of stabilization reagent was added to each sample. The absorbance at 635 nm was then determined using a Tecan M200 Infinite plate reader. The rate of GmhA was determined by comparison to a phosphate standard curve ranging from 0–70 μM phosphate, and by subtracting the background rate from negative controls containing no GmhA. All reactions were performed in triplicate, and a total of eight substrate concentrations were tested for each sample. The kinetic parameters of each GmhA preparation were determined by fitting the data to Equation 1, which describes a cooperative model, or the Michaelis-Menten equation (equation 2), using GraphPad Prism version 6.0.2 (GraphPad Software, La Jolla, CA). Equations including a product release term were tested: these did not alter the kinetic parameters, and were rejected according to the Akaike Information Criteria.

1 $$v=\,{k}_{cat}\,\frac{{E}_{t}{(\frac{[S]}{{K}_{1/2}})}^{n}}{1+\,{(\frac{[S]}{{K}_{1/2}})}^{n}}$$

2 $$v=\,{k}_{cat}\,\frac{{E}_{t}[S]}{{K}_{M}+[S]}$$

The co-ordinates and structure factors for the structures described in this paper have been submitted to the Protein Data Bank with accession numbers 5LU7, 5LU6, 5LU5 and 5LTZ for the D61A, H64Q, E68Q and Q175E structures respectively.

## Computational studies

### Global hydrogen bond analysis

Samples were prepared for computational analysis by setting all residues with alternate conformations to the most favored conformation using PHENIX. Structure with S7P molecules in defined locations were prepared by adding S7P molecules as appropriate from the Q175E structure to the wild-type structure (PDB ID: 2XBL). Explicit hydrogens were added to the structure, and the structure optimized, using the online version of YASARA^39^. Ten variants of each structure were produced by adding random shifts with an average of 0.058 Å to the heavy atoms using PDBSET^40^, and adding hydrogens using YASARA. Hydrogen bonding network analysis was performed using Proflex^29^. Hydrogen bond dilution was performed using default settings. The strength of the hydrogen bonding network was determined automatically using HETHER^41^. HETHER aberrantly assessed approximately one-quarter of the networks with GmhA added to active sites A and B; these replicates were removed and replaced with additional replicates.

### Quantum mechanical calculations

A total of five model systems were set up. Model 1 and Model 2 are single active site models. Model 1 contains the zinc and the coordinating H64, E68, Q175 and H183 (unliganded). Model 2 is Model 1 with addition of the substrate S7P (substrate-bound). Models 3, 4 and 5 are all paired active site models, in which D61 from the two subunits (labelled as *Sub_A* and *Sub_B*) and a water molecule link the paired active sites. Specifically, Model 3 contains two unliganded paired active sites; Model 4 contains one unliganded active site and one substrate-bound active site S7P, while Model 5 includes two substrate-bound paired active. In addition, two water molecules are incorporated into Model 5 (labelled as Model 5^*water*^) to mimic water molecule or the sidechain of S58, which are within hydrogen bonding distances to both D61 and Q175 during our preliminary molecular dynamics calculations. The initial structures of these models were taken from the apo enzyme (PDB ID: 2X3Y) and the Q175E mutant. Only the sidechains of the active site residues and the aldehyde and ketone moiety at position 1 and 2 of S7P were kept in the models. The methyl groups (−CH_3_) of the sidechains of active site residues were fixed during QM optimisation, so that the residues didn’t move too far away from their positions in the crystal structures. The models were optimized at M05 2X/6–31 + G* level, using program G09^42^ to local minima and verified by frequency calculations. M05 2X level has been shown to be able to model the geometry and binding energy of zinc complexes well^43,44^. Zinc’s electron accepting ability (Lewis acidity) was estimated by the condensed Fukui indices *f* ^+^_zn_

$${{f}^{+}}_{Zn}\approx {q}_{zn}(n+1)-{q}_{zn}(n)$$

where *q*_zn_(*n*) and *q*_zn_(*n* + 1) represent, respectively, zinc’s charge in the model system (containing *n* electrons) and its charge upon adding one electron (*n* + 1) to the model systems (one Zn^2+^ in Model 1) and two electrons (*n* + 2) (two Zn^2+^ in Model 3)^45,46^. The natural population analysis was used to calculate the charge of zinc ions at the M05 2X/6–31 + G* level.

### Data Availability

The research materials supporting this publication can be accessed by contacting the corresponding author.

## Electronic supplementary material

Supplementary figures

dataset 1
