# Supplementary material for: A half-site multimeric enzyme achieves its cooperativity without conformational changes
Source: Sci Rep. 2017 Nov 28;7:16529. doi: 10.1038/s41598-017-16421-2 (PMC5705639; doi:10.1038/s41598-017-16421-2)
Supplement: Supplementary file 1 — Supplementary figures [file 41598_2017_16421_MOESM1_ESM.pdf]

**Vivoli, Pang and Harmer: A half-site multimeric enzyme achieves its cooperativity without conformational changes**

**Supplementary information**

**Supplementary Figure 1: Catalytic action of sedoheptulose-7-phosphate isomerase (GmhA). a:** Reaction catalyzed by GmhA. Sedoheptulose-7-phosphate (left, S7P) is isomerized into D-*glycero*-D-*manno*-heptopyranose 7-phosphate (right, M7P). The oxygen atoms that are involved in the reaction are colored red (O1), blue (O2) and brick red (O5) respectively to highlight the alteration in the molecule. **b:** GmhA interactions with product. The product M7P (D-*glycero*-D-*manno*-heptopyranose-7-phosphate) interacts widely with the enzyme active site. In particular, D98 (red arrow) makes hydrogen bonds to both $O^1$ and $O^5$. D98 is expected to therefore contribute strongly to the ring cyclization of the M7P product.

**a**

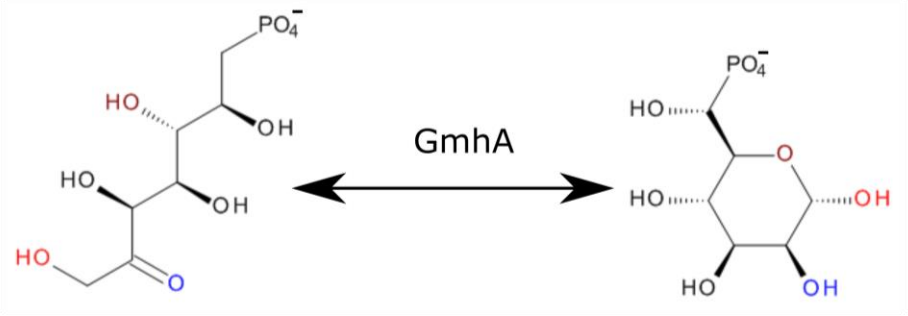


**b**

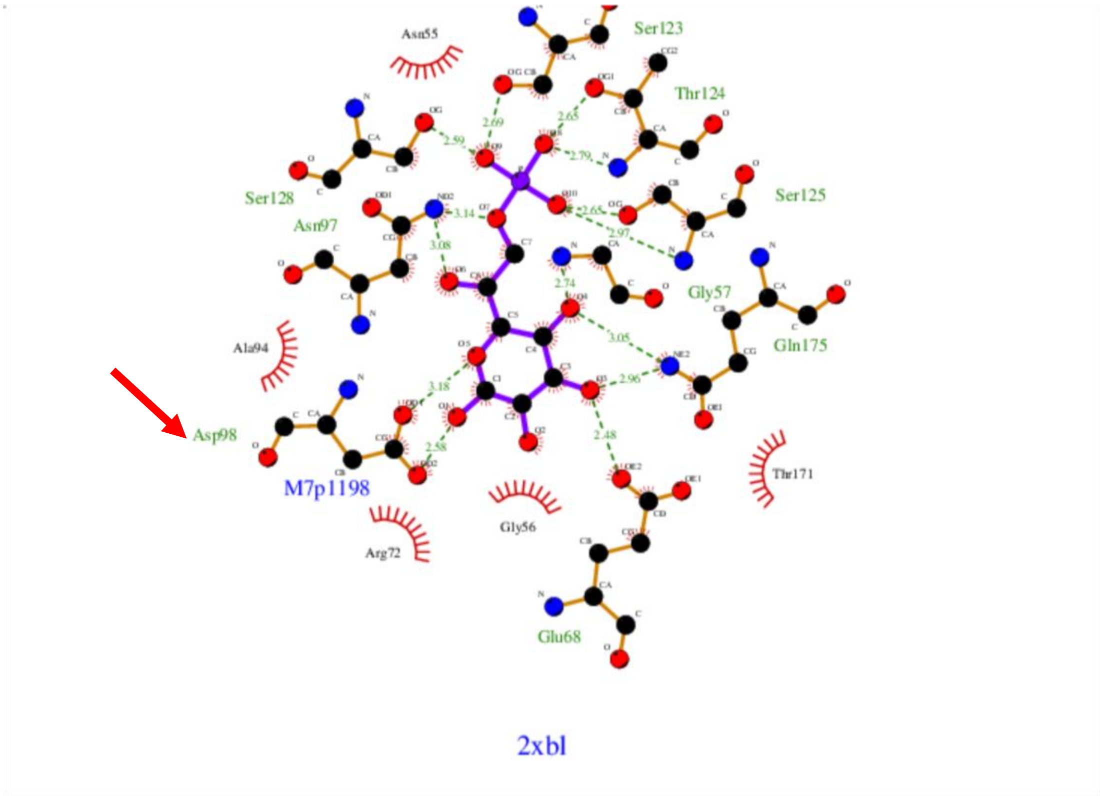

**Supplementary Figure 2: A solvent channel links pairs of active sites in GmhA. a:** Overview of the structure of GmhA. Pairs of active sites are located in close proximity to one another at the top (red box) and bottom of the molecule. **b:** Close-up, cut away view of the water filled channel linking pairs of active sites. The only hydrophilic side chains in this channel are H64 (coordinating to the catalytic zinc) and D61. Images generated using PyMOL from PDB ID 2XBL [1]. Protein backbone shown as cartoon; product, H64 and D61 as sticks; zinc as spheres. Colors: GmhA chain A, sky blue; chain B, white; chain C, green; chain D, brick red; substrate S7P carbon, cyan; product M7P carbon, yellow; oxygen, red; nitrogen, blue; phosphorus, orange; zinc, purple sphere.

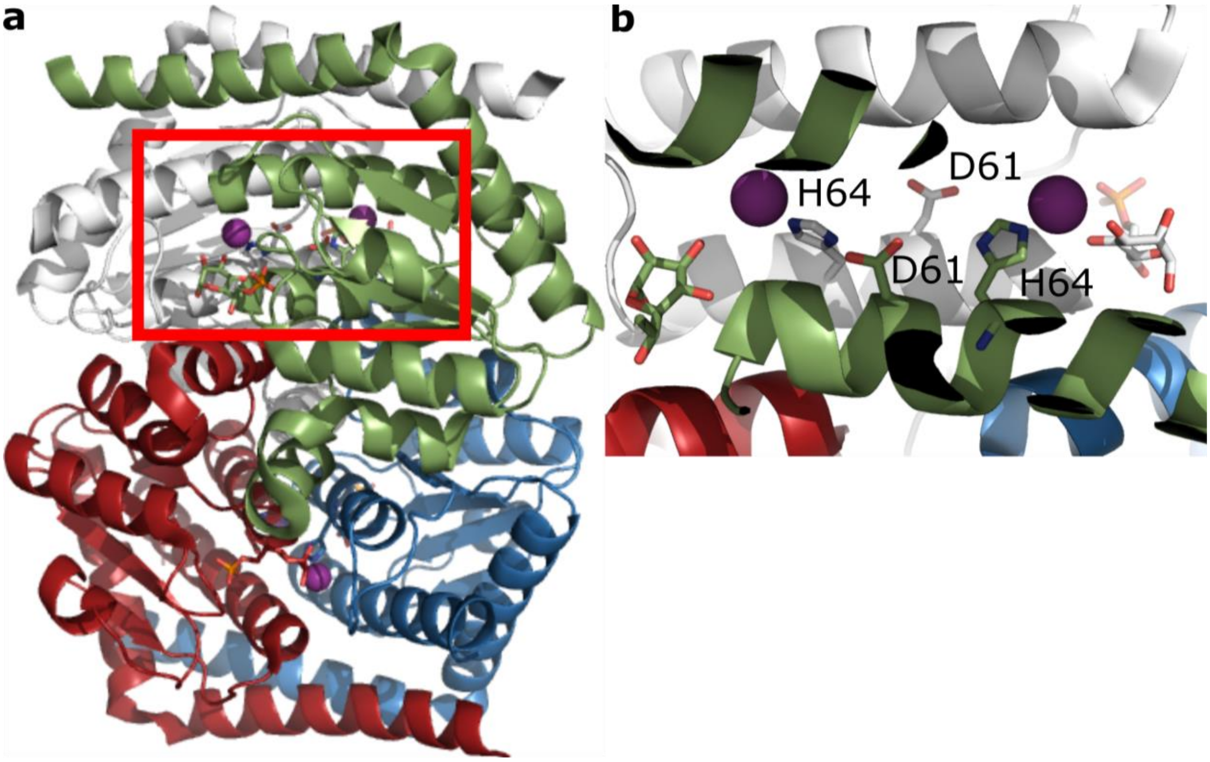

**Supplementary Figure 3: Purification of GmhA tetramers with varying numbers of active sites.** Two versions of GmhA were co-expressed – 6*His-GmhA E115K (a surface polymorphism seen between the standard K96243 and 1710b strains of *B. pseudomallei*), and GmhA D98N (lacking a critical substrate binding and catalytic residue). The protein was purified by IMAC and size exclusion chromatography; and then further purified over an anion exchange (Q) column, giving the elution profile shown below. The more positively charged E115K mutant is expected to elute earlier than the Four peaks were modelled as Gaussian curves, with the height and width of each peak fitted to the observed data using least-squares fitting using Excel (green, purple, cyan, orange lines respectively). The overall fit of the model (light blue) to the data (blue) is very good. The final fractions for analysis were selected for peaks 1, 2 and 4 to contain less than 1% contaminating material. No fraction contained less than 10% contaminating material for peak 3, so this was not further studied. Mass spectrometry showed that peak 2 contained approximately nine times more of the larger 6*His-GmhA E115K per molecule of GmhA D98N than peak 4. This implies that these represent the 3:1 and 1:3 ratios of 6*His-GmhA E115K and GmhA D98N respectively, as expected from the ion exchange result.

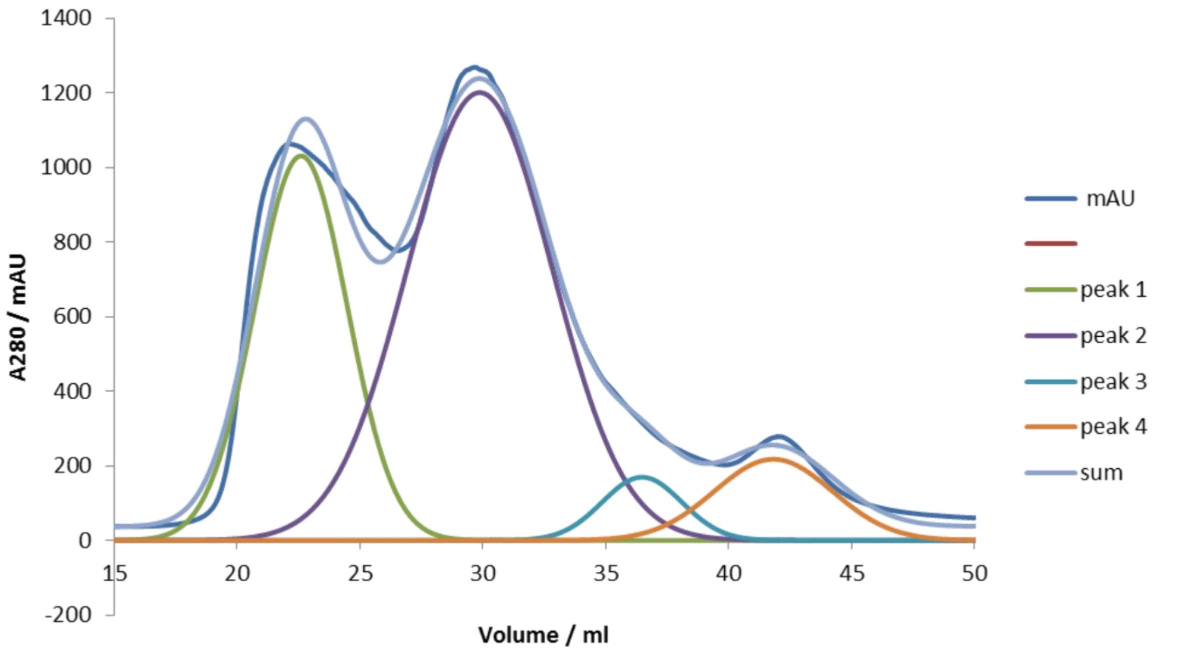

**Supplementary Figure 4: GmhA variants with differing numbers of active sites all show cooperativity in their activity.** Assay of GmhA variants containing four (black), three (red) or one (blue) active sites all show positive cooperativity in the same manner as wild-type. Best fit cooperative models are shown in full lines; best fit Michaelis-Menten models are shown in dashed lines. Each dataset shows shows positive cooperativity (>99% confidence in cooperative model in all cases, Akaike's information criterion implemented in Graphpad; h = 2.4 ± 0.3, 2.0 ± 0.2, 2.3 ± 0.4 respectively for samples containing four, three and one intact active site). All data shown are biological triplicates, with error bars showing ± SEM.

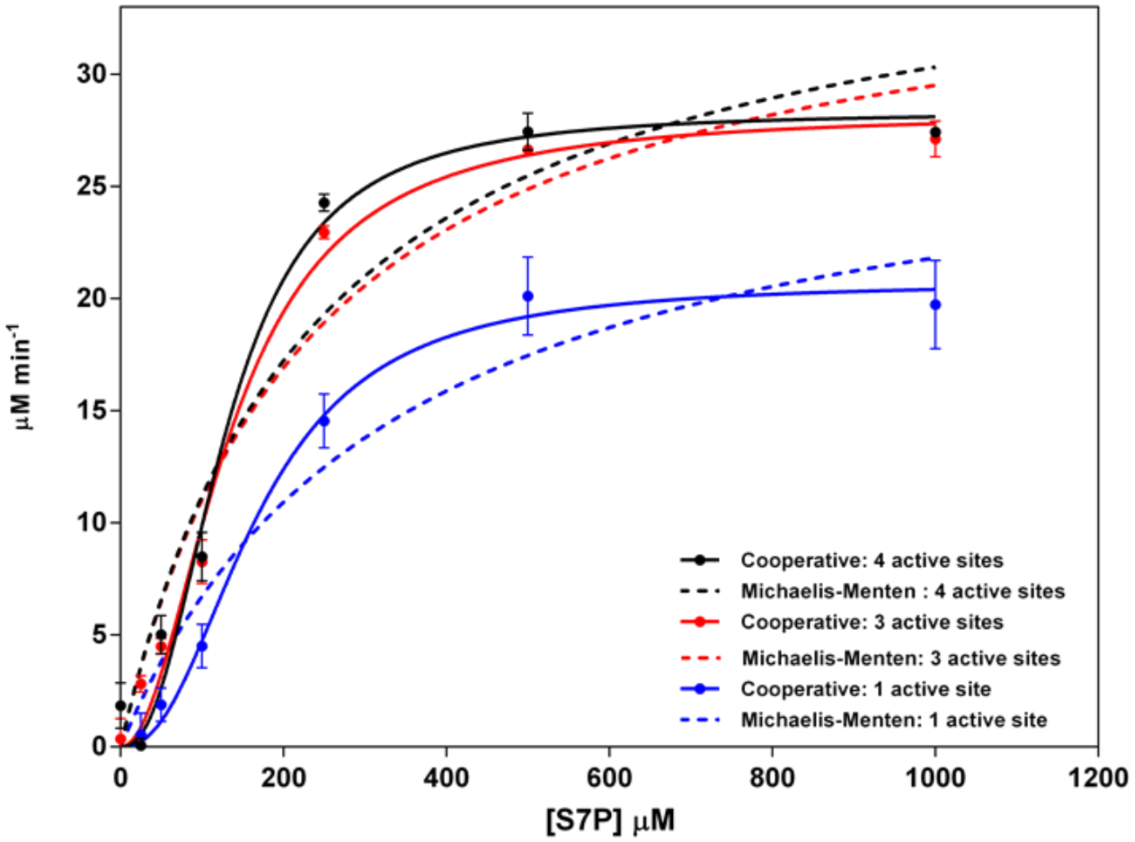

**Supplementary Figure 5: There is no conformational change between substrate and product bound GmhA.** The structures of substrate (PDB ID: 5LTZ, magenta, this study) and product (PDB ID: 2XBL, cyan [1]) bound GmhA were superimposed. There is no significant alteration in the structure, with an RMS deviation of 0.189 Å between the 4,967 aligned atoms in the structures. Figure generated using PyMOL. Main chain shown as cartoon, and substrate/product as sticks. Colors: substrate bound cartoon, magenta; product bound cartoon, cyan; substrate carbon, purple; product carbon, yellow; oxygen, red; phosphorus, orange.

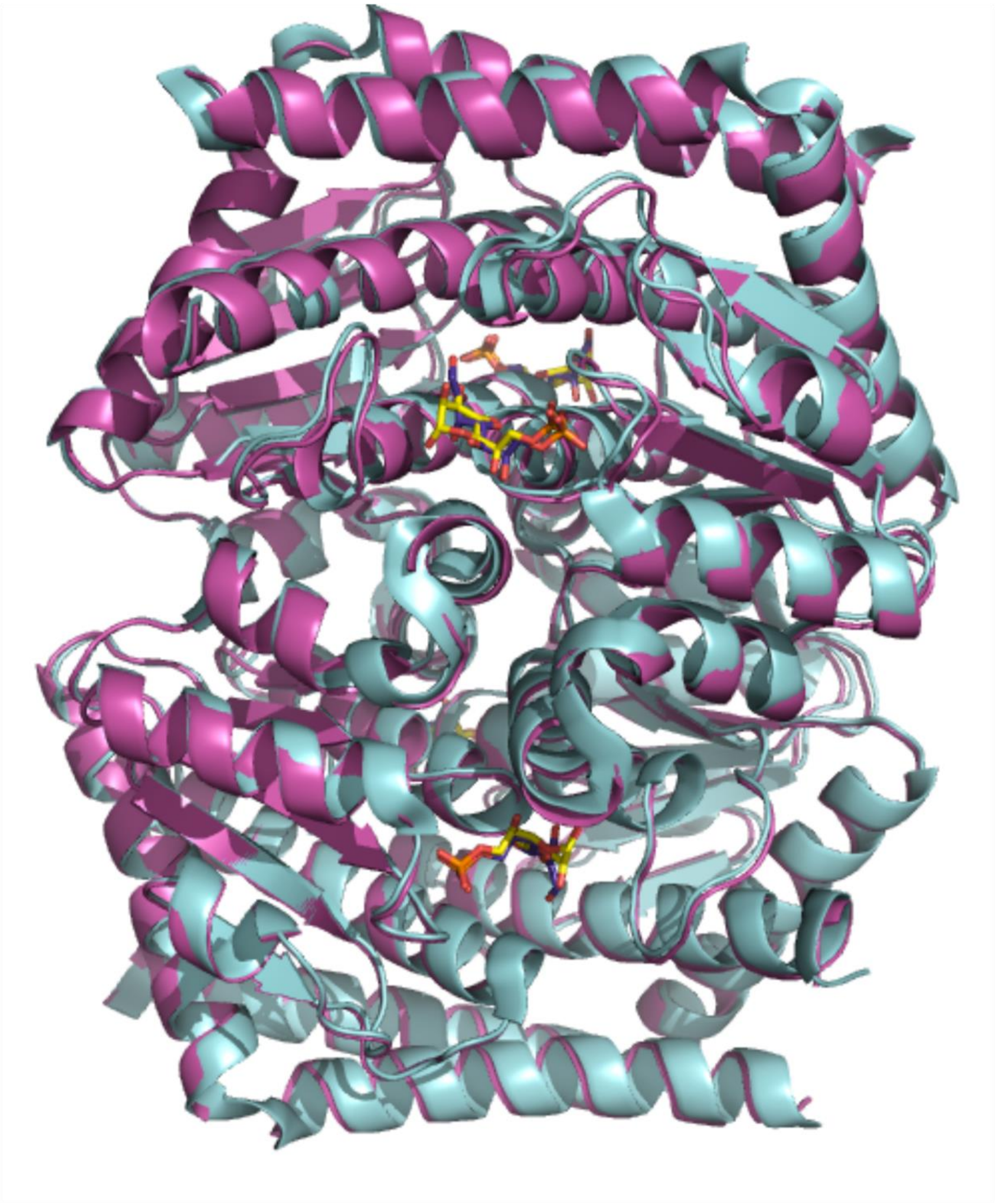

**Supplementary Figure 6: The structure of GmhA H64Q shows substrate in the active site.** The location of the substrate reflects that of the substrate observed in the E68Q mutant. Similar density is observed in all four active sites. Protein is shown as cartoon, and ligand binding residues as sticks. Density shown as red netting is $2F_o$-$F_c$, contoured at 1σ. Colors: GmhA chain A, sky blue; chain B, white; chain D, brick red; substrate S7P carbon, green; oxygen, red; nitrogen, blue; phosphorus, orange. Figure generated using PyMOL.

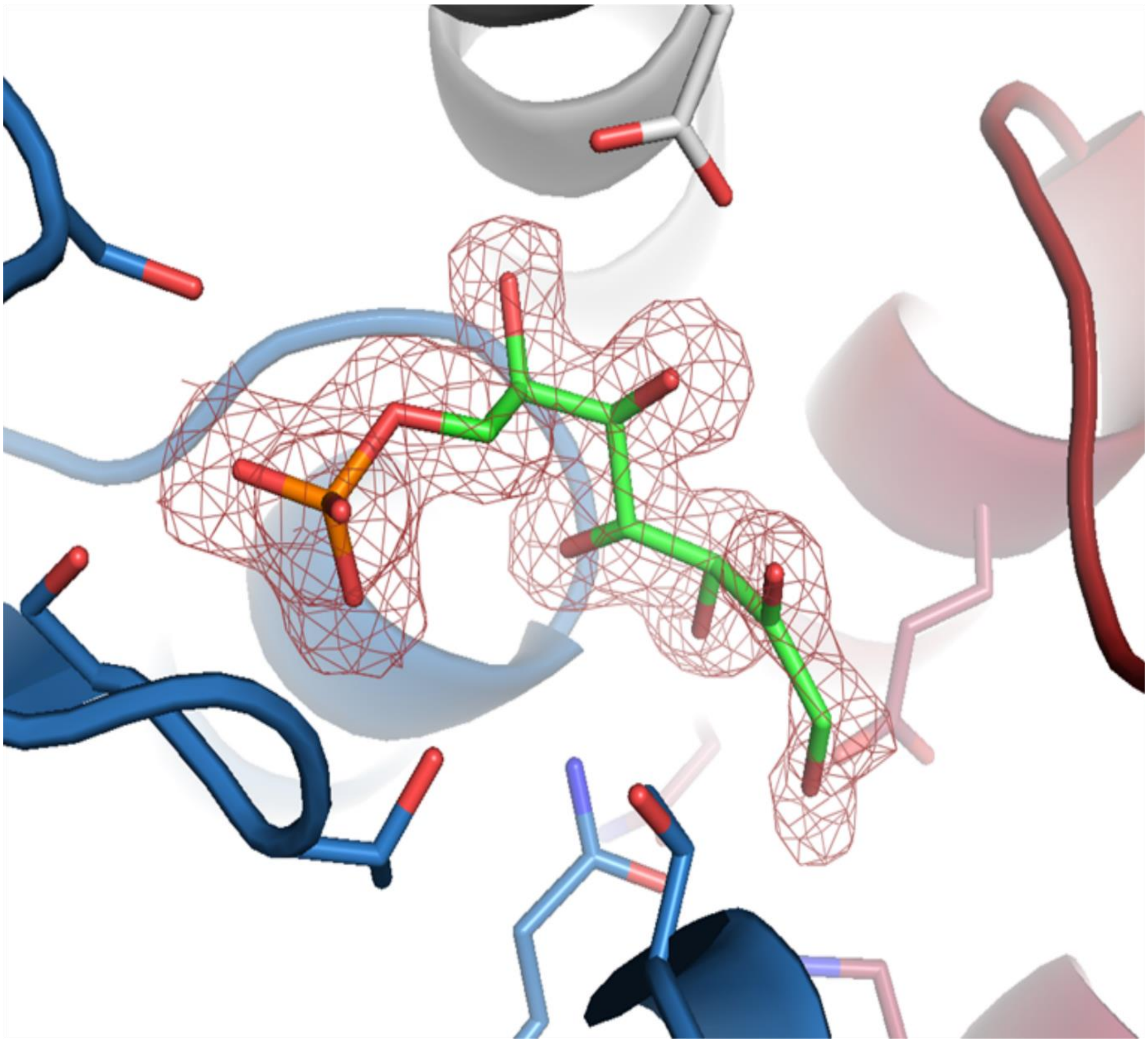

**Supplementary Figure 7: Hydrogen bond dilution of GmhA in complex with substrate**. The hydrogen-bonding network of GmhA was assessed in the absence of ligands (**a**), or the presence of one S7P molecule (**b**), two S7P molecules on the same face of GmhA (**c**), and two S7P molecules on opposite faces of GmhA (**d**). Hydrogen bonding dilution using PROFLEX [2] was used to highlight strongly and weakly bonded regions of the protein. The output from the hydrogen bonding dilution is shown, with sections of protein bonded together shown in the same color.

In the ligand free state (**a**), the major hydrogen bonding network shows rigidity until hydrogen bonds weaker than 1.9 kcal/mol are broken (red arrow in each panel indicates the point where the major hydrogen bonding network loses integrity). Some external helices break free from this network at lower energies. Binding of one S7P molecule (**b**) causes the network to become weaker, losing rigidity at an energy 0.3 kcal/mol lower. Binding of two molecules of S7P (**c**) to the same face of GmhA (*"cis"* complex) causes the network to lose rigidity at even lower energy (~0.1 kcal/mol earlier). When two S7P molecules bind to opposite faces of GmhA (**d**; "*trans*" complex), in contrast, the network shows much greater stability, maintaining rigidity for another 0.4 kcal/mol beyond the unliganded GmhA.

a:

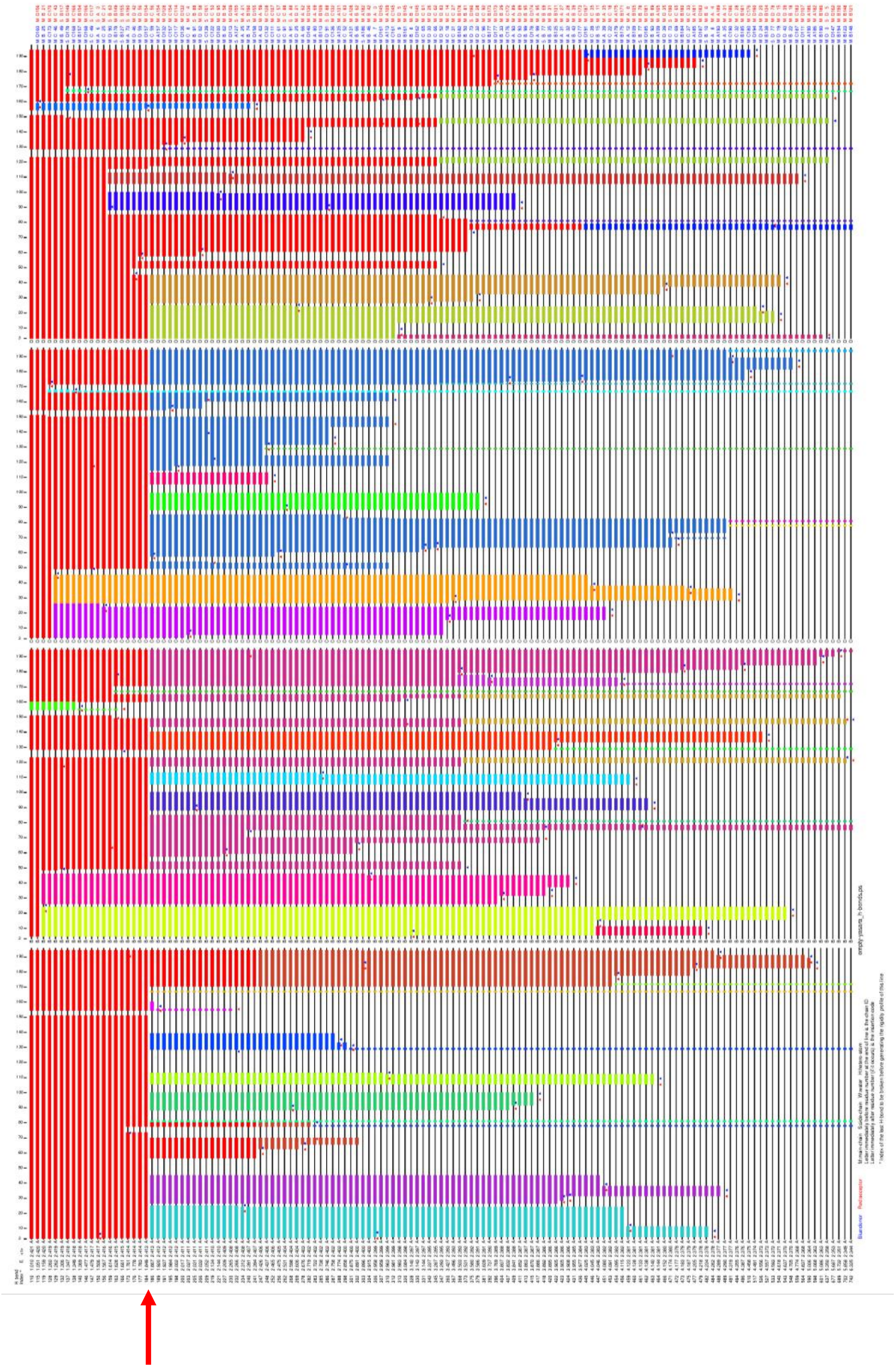

**b:**

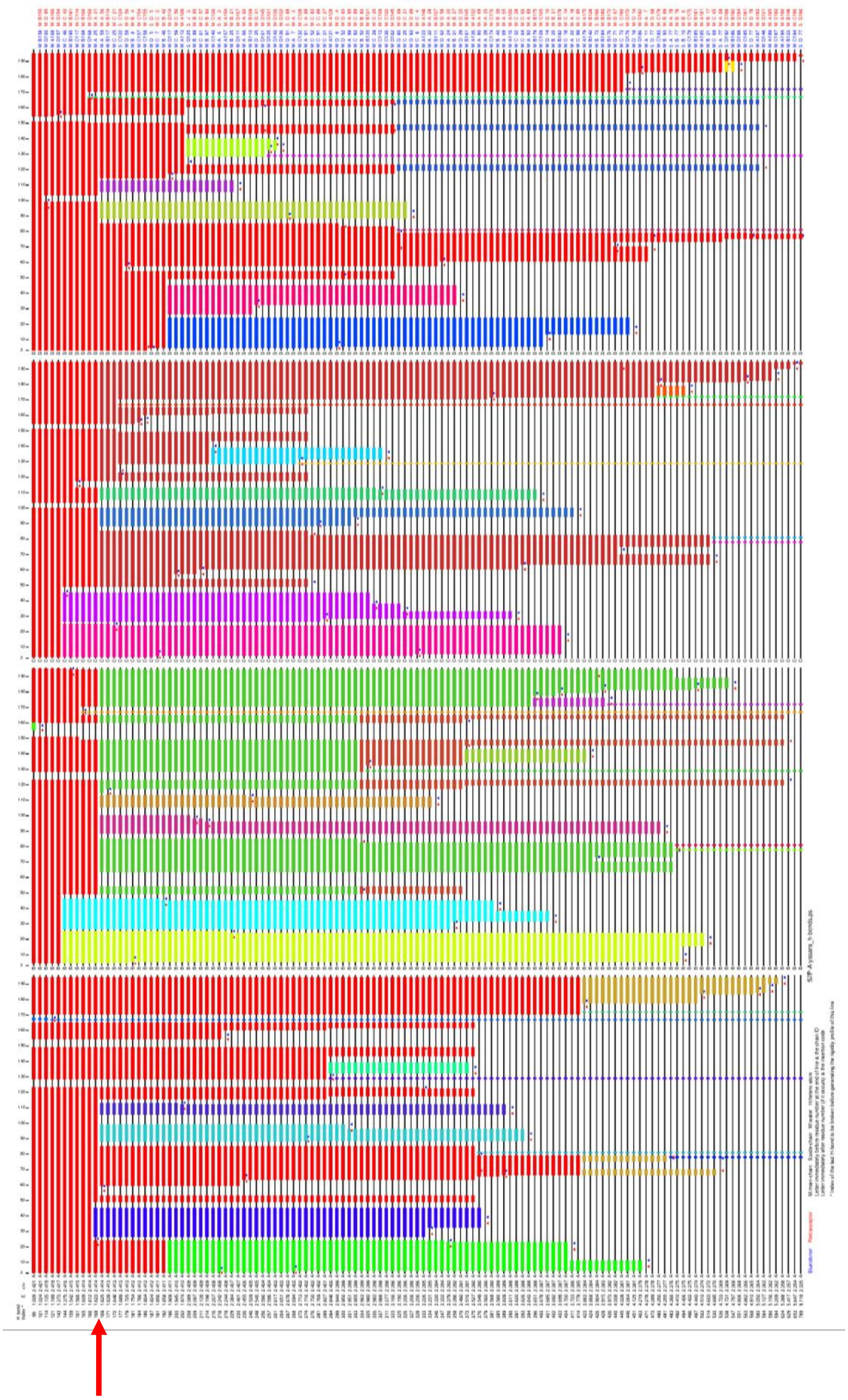

**c:**

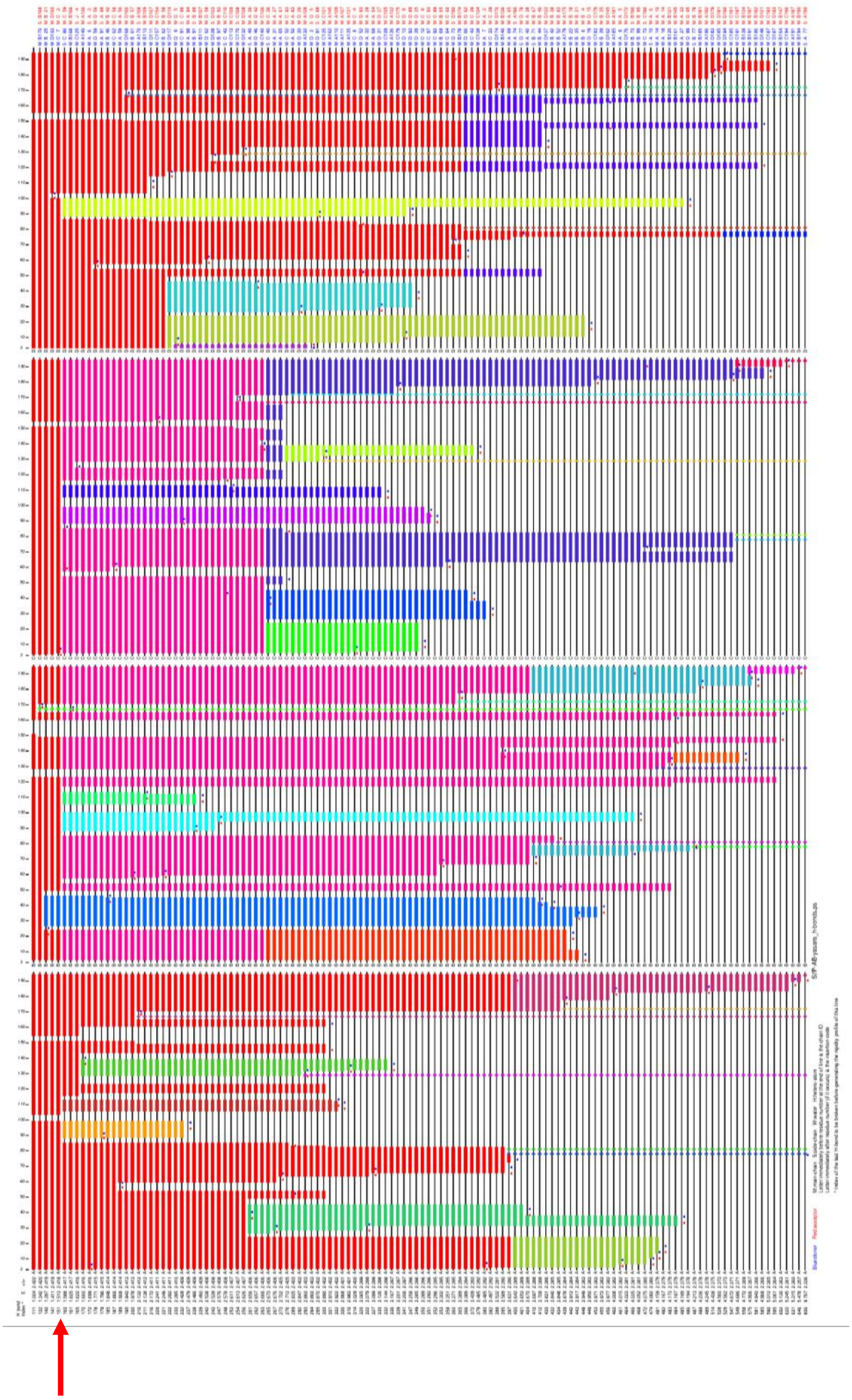

d:

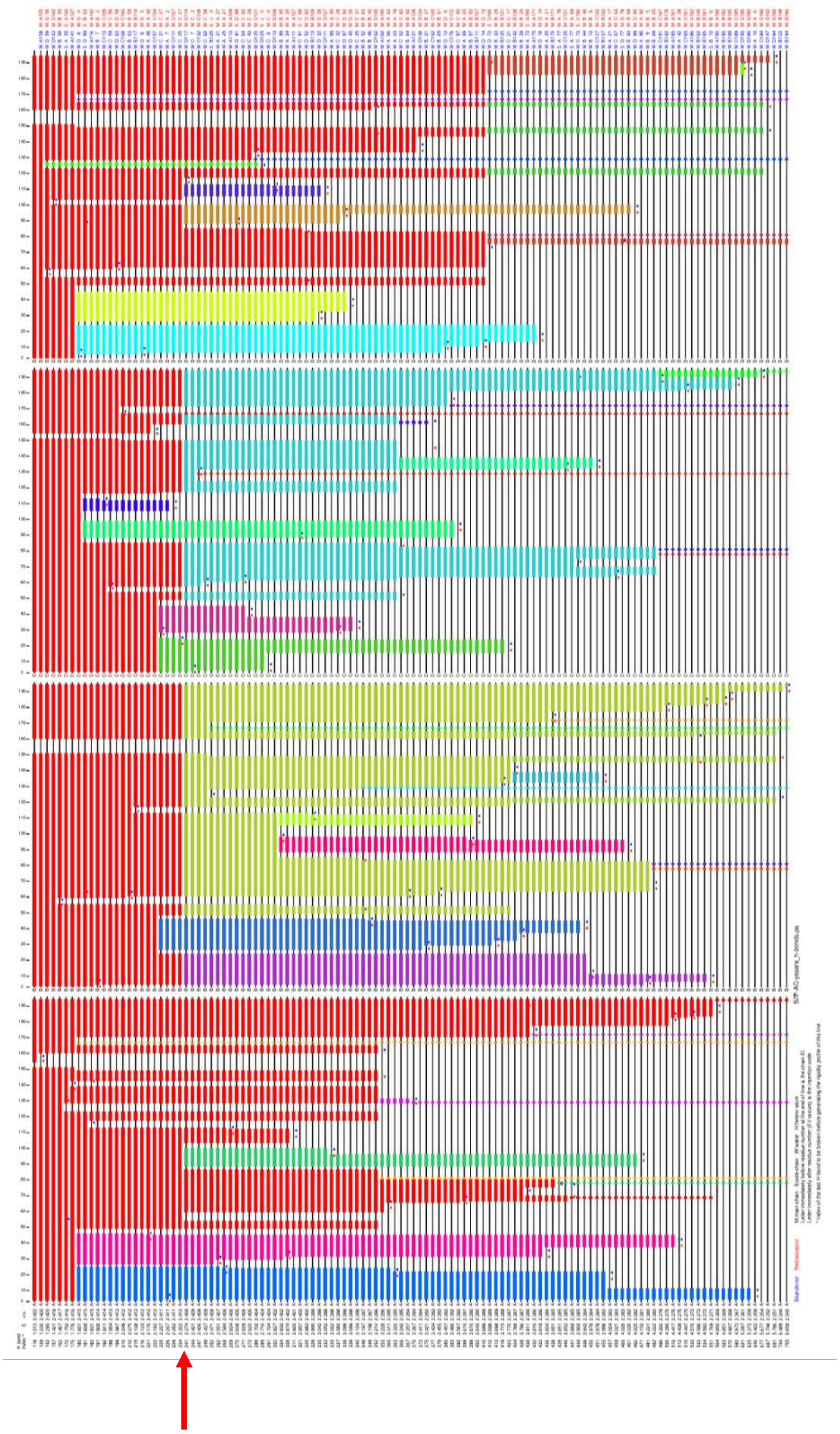

**Supplementary Table 1: Crystallographic data collection and refinement statistics.**

| Mutant | D61A | H64Q | E68Q | Q175E |
|---|---|---|---|---|
| **PDB ID** | 5LU7 | 5LU6 | 5LU5 | 5LTZ |
| **Crystallisation conditions** | 50 mM HEPES pH 7.5, 10% (w/w) PEG 3350, 5 mM each $ZnCl_2$, $MgCl_2$, KCl, $CaCl_2$. | 50 mM HEPES pH 7.5, 8.5% (w/w) PEG 3350 | 50 mM HEPES pH 7.0, 7% (w/w) PEG 3350 | 50 mM MES pH 6.5, 8.5% (w/w) PEG 3350 |
| **Cryoprotectant solution and soaking time.** | 5 mM S7P, 10% (w/w) PEG 3350, 20% (v/v) PEG 400; 30 min | 5 mM S7P, 8.5% (w/w) PEG 3350, 21.5% (v/v) PEG 400; 5 min. | 25 mM S7P, 35% (v/v) PEG 400; 65 min. | 25 mM S7P, 35% (v/v) PEG 400; 55 min. |
| **Data Collection** | | | | |
| Wavelength (Å) | 0.97950 | 0.97950 | 0.97950 | 0.97950 |
| Space group | $P2_12_12_1$ | $P2_12_12_1$ | $P2_12_12_1$ | $P2_12_12_1$ |
| Cell dimensions | | | | |
| *a*, *b*, *c* (Å) | 73.2, 83.68, 126.10 | 73.61, 84.61, 127.09 | 73.20, 83.81, 126.78 | 72.55, 84.47, 126.68 |
| $\alpha, \beta, \gamma$ (°) | 90, 90, 90 | 90, 90, 90 | 90, 90, 90 | 90, 90, 90 |
| Resolution (Å)* | 50.33-1.92 (1.97-1.92) | 63.70-1.67 (1.76-1.67) | 63.40-1.55 (1.59-1.55) | 26.25-1.67 (1.71-1.67) |
| $R_{pim}$ * | 0.035 (0.310) | 0.057 (0.237) | 0.028 (0.302) | 0.050 (0.569) |
| $I / \sigma I$ * | 17.3 (2.5) | 7.4 (2.2) | 17.5 (2.7) | 7.6 (1.0) |
| Completeness (%) * | 99.8 (99.7) | 88.6 (89.3) | 99.5 (99.0) | 99.9 (99.9) |
| Redundancy * | 5.5 (5.6) | 6.4 (6.2) | 6.3 (5.2) | 5.6 (5.7) |
| **Refinement** | | | | |
| Resolution (Å) | 37.54-1.92 | 42.31-1.67 | 63.40-1.55 | 26.25-1.67 |
| No. reflections | 59262 | 73739 | 112695 | 90441 |
| $R_{work}$ / $R_{free}$ (%) | 17.03/21.48 | 19.10/22.60 | 16.11/18.72 | 20.75/24.47 |
| No. atoms | | | | |
| Protein | 5733 | 5763 | 5795 | 5750 |
| Ligand/ion | 123 | 120 | 191 | 130 |
| Water | 557 | 699 | 813 | 380 |
| *B*-factors | | | | |
| Protein | 25.96 | 20.02 | 18.36 | 33.82 |
| Ligand/ion | 34.3 | 34.4 | 28.93 | 40.45 |
| Water | 32.31 | 31.05 | 30.82 | 39.57 |
| R.m.s. deviations | | | | |

| | | | | |
|---|---|---|---|---|
| Bond lengths (Å) | 0.007 | 0.019 | 0.006 | 0.007 |
| Bond angles (°) | 1.047 | 1.792 | 1.031 | 1.059 |
| Ramachandran favoured/ allowed/outlier (%) † | 97.8/2.2/0 | 98.2/1.7/0.1 | 98.3/1.7/0 | 97.7/2.0/0.3 |

*Values in parentheses are for highest-resolution shell. † Calculated using RAMPAGE (http://mordred.bioc.cam.ac.uk/~rapper/rampage.php).

**Supplementary Table 2: Summary of the distances and angles for zinc coordination which were obtained at M05 2X/6-31+G*.** Points where no value is appropriate for the model are indicated by a dash. The values for the second site of the models with two active sites and two ligands (Model 5 and Model $5^{water}$) that are at variance with other liganded sites are shown in bold and red.

| | | Models | | | | | | | | | |
|---|---|---|---|---|---|---|---|---|---|---|---|
| **Distances (Å)** | 2XBL | 1 | 2 | $3^A$ | $3^B$ | $4^A$ | $4^B$ | $5^A$ | $5^B$ | $5\_wat^A$ | $5\_wat^B$ |
| Zn-Glu68OE2 | 1.95 | 2.06 | 1.97 | 2.00 | 1.97 | 2.01 | 1.99 | 2.02 | 1.98 | 1.97 | 1.97 |
| Zn-His183NE2 | 2.02 | 2.03 | 2.12 | 2.07 | 2.05 | 2.16 | 2.06 | 2.13 | 2.04 | 2.12 | 2.07 |
| Zn-His64NE2 | 2.04 | 2.04 | 2.17 | 2.03 | 2.05 | 2.13 | 2.03 | 2.11 | 2.01 | 2.14 | 2.01 |
| Zn-Gln175OE2 | 2.04 | 2.21 | 2.05 | 2.14 | 2.04 | 2.06 | 2.12 | 2.02 | 2.00 | 2.01 | 2.00 |
| Zn-S7PO2(C=O) | - | - | 2.48 | - | - | 2.66 | - | 2.69 | **3.50** | 2.67 | 2.63 |
| Zn-S7PO1(OH) | - | - | 2.16 | - | - | 2.19 | - | 2.16 | **3.78** | 2.24 | **3.14** |
| **Angles (°)** | | | | | | | | | | | |
| Glu68OE2-Zn-His183NE2 | 119.44 | 128.9 | 91.3 | 124.0 | 121.7 | 88.8 | 114.5 | 90.8 | 109.0 | 88.3 | 97.7 |
| His183NE2-Zn-His64NE2 | 111.79 | 116.3 | 107.1 | 109.1 | 106.1 | 110.7 | 110.4 | 102.3 | 110.1 | 108.2 | 113.6 |
| His64NE2-Zn-Glu68OE2 | 110.96 | 108.7 | 83.7 | 113.3 | 111.6 | 88.4 | 109.3 | 85.3 | 112.8 | 91.5 | 114.9 |
| Gln175OE1-Zn-Glu68OE1 | 111.91 | 110.9 | 177.1 | 110.6 | 115.0 | 169.4 | 128.5 | 163.4 | 121.0 | 156.7 | 132.8 |
| Gln175OE1-Zn-His183NE2 | 98.76 | 91.7 | 91.6 | 96.8 | 97.2 | 97.1 | 92.3 | 105.0 | 100.7 | 108.8 | 91.4 |
| Gln175OE1-Zn-His64NE2 | 101.96 | 91.25 | 94.9 | 99.2 | 102.9 | 97.6 | 99.9 | 96.1 | 102.4 | 97.7 | 103.2 |
| S7PO1-ZN-S7PO2 | - | - | 70.3 | - | - | 67.1 | - | 66.9 | **44.5** | 67.1 | **57.7** |
| S7PO2-Zn-His64NE2 | - | - | 88.2 | - | - | 90.0 | - | 99.3 | **135.1** | 92.3 | 98.7 |
| His183NE2-Zn-S7PO1 | - | - | 94.4 | - | - | 91.8 | - | 90.6 | **77.7** | 92.3 | 90.1 |

**Supplementary Table 3: Cartesian coordinates (in xyz format) of all optimized models obtained at M05 2X/6-31+G* level.**

Model_1

41

| | | | |
|---|---|---|---|
| C | -35.189001 | 68.950999 | -4.728000 |
| H | -35.235564 | 70.009238 | -4.476826 |
| H | -35.186251 | 68.382039 | -3.796088 |
| H | -36.092573 | 68.696296 | -5.285476 |
| C | -33.961248 | 68.688364 | -5.528333 |
| N | -33.619808 | 67.441263 | -6.012967 |
| H | -34.156626 | 66.596209 | -5.891478 |
| C | -32.953682 | 69.512503 | -5.939636 |
| H | -32.830845 | 70.571160 | -5.785228 |
| C | -32.452336 | 67.538661 | -6.678905 |
| N | -32.021439 | 68.783525 | -6.650274 |
| H | -31.958412 | 66.714012 | -7.164068 |
| C | -29.742997 | 72.440985 | -4.574013 |
| H | -30.036566 | 73.424970 | -4.930440 |
| H | -30.394740 | 72.157549 | -3.744543 |
| H | -28.714145 | 72.439092 | -4.220527 |
| C | -29.932289 | 71.422858 | -5.688304 |
| O | -30.823441 | 71.609615 | -6.557695 |
| O | -29.228234 | 70.345789 | -5.741554 |
| C | -28.567006 | 65.393024 | -8.102996 |
| H | -28.027979 | 64.698542 | -7.461761 |
| H | -29.550458 | 64.991730 | -8.342375 |
| H | -28.016592 | 65.522737 | -9.036095 |
| C | -28.740629 | 66.762742 | -7.454376 |
| O | -29.527409 | 67.611639 | -7.930508 |
| N | -28.022111 | 67.036389 | -6.364801 |
| H | -28.098545 | 67.950972 | -5.932400 |
| H | -27.371507 | 66.374922 | -5.973542 |
| C | -31.149996 | 72.623992 | -11.993991 |
| H | -31.482297 | 73.498329 | -11.437154 |
| H | -30.308907 | 72.922224 | -12.622691 |
| H | -31.969305 | 72.300145 | -12.638585 |
| C | -30.756124 | 71.545454 | -11.035592 |
| N | -30.288661 | 70.311874 | -11.449202 |
| H | -30.159748 | 70.024804 | -12.407362 |
| C | -30.752790 | 71.477416 | -9.669646 |
| H | -31.038794 | 72.220276 | -8.943866 |
| C | -30.027435 | 69.555005 | -10.366924 |
| N | -30.297719 | 70.234368 | -9.269314 |
| H | -29.658483 | 68.543958 | -10.401789 |
| Zn | -30.287297 | 69.601058 | -7.340796 |

Model_2

52

| | | | |
|---|---|---|---|
| C | 1.646333 | 5.125194 | -0.478484 |
| H | 2.303546 | 5.687941 | 0.187093 |
| H | 2.190736 | 4.920317 | -1.398750 |
| H | 0.784591 | 5.748218 | -0.725236 |
| C | 1.214183 | 3.829108 | 0.141970 |
| N | 0.504006 | 3.762964 | 1.326845 |
| H | 0.225099 | 4.550953 | 1.890464 |

| | | | |
|---|---|---|---|
| C | 0.224656 | 2.468653 | 1.593562 |
| N | 0.717610 | 1.687236 | 0.655413 |
| H | -0.341824 | 2.144514 | 2.451792 |
| C | 1.336647 | 2.524321 | -0.253785 |
| H | 1.797231 | 2.131560 | -1.143967 |
| C | 0.061114 | 0.989020 | -3.908752 |
| H | 1.043316 | 1.459990 | -3.819479 |
| H | -0.688337 | 1.771772 | -3.797529 |
| H | -0.017352 | 0.520242 | -4.887303 |
| C | -0.083937 | -0.009682 | -2.808077 |
| O | -0.243141 | 0.509952 | -1.657719 |
| O | -0.020715 | -1.240512 | -3.035585 |
| C | 1.210662 | -1.416023 | 4.163013 |
| H | 2.100193 | -1.266422 | 4.772985 |
| H | 0.949216 | -2.474246 | 4.156887 |
| H | 0.374915 | -0.871322 | 4.603705 |
| C | 1.378165 | -0.960930 | 2.747789 |
| O | 0.432597 | -1.128886 | 1.953783 |
| N | 2.526838 | -0.391553 | 2.386511 |
| H | 2.654957 | -0.083807 | 1.429738 |
| H | 3.276870 | -0.262159 | 3.045006 |
| C | -5.606304 | 0.051738 | -0.575890 |
| H | -6.275969 | 0.446139 | 0.190801 |
| H | -6.055236 | -0.848265 | -1.000377 |
| H | -5.523426 | 0.793705 | -1.368273 |
| C | -4.244609 | -0.233581 | -0.018949 |
| N | -4.025155 | -1.133127 | 1.007645 |
| H | -4.734312 | -1.676889 | 1.474246 |
| C | -2.700911 | -1.185412 | 1.266053 |
| N | -2.048227 | -0.365525 | 0.469626 |
| H | -2.252543 | -1.823533 | 2.009279 |
| C | -2.997415 | 0.231541 | -0.337943 |
| H | -2.710995 | 0.937201 | -1.099213 |
| Zn | 0.038515 | -0.302934 | 0.115237 |
| C | 3.807875 | -2.482586 | -1.569633 |
| H | 3.993283 | -3.510730 | -1.253038 |
| H | 3.698024 | -2.487826 | -2.658299 |
| H | 4.637871 | -1.835386 | -1.295192 |
| C | 2.514327 | -1.968502 | -0.983574 |
| O | 2.368030 | -0.825604 | -0.566347 |
| C | 1.349015 | -2.939039 | -0.915477 |
| H | 1.522523 | -3.598015 | -0.057776 |
| H | 1.343115 | -3.560318 | -1.815783 |
| O | 0.117667 | -2.280566 | -0.754232 |
| H | -0.112108 | -1.939186 | -1.702379 |

Model_3
99

| | | | |
|---|---|---|---|
| C | -39.563653 | 68.542507 | -7.211120 |
| H | -38.581129 | 68.981150 | -7.393227 |
| H | -39.666336 | 68.381661 | -6.135539 |
| H | -40.351606 | 69.209201 | -7.557383 |
| C | -39.651419 | 67.163738 | -7.922992 |
| O | -38.689092 | 66.372410 | -7.751394 |
| O | -40.691133 | 66.928147 | -8.608075 |
| C | -35.188897 | 68.950871 | -4.727919 |
| H | -34.906866 | 69.756980 | -4.051673 |
| H | -35.352393 | 68.044388 | -4.142089 |
| H | -36.135888 | 69.211426 | -5.204893 |

| | | | |
|---|---|---|---|
| C | -34.116462 | 68.751864 | -5.735894 |
| N | -34.163090 | 67.815656 | -6.741164 |
| H | -34.895793 | 67.078210 | -6.894038 |
| C | -32.927903 | 69.407075 | -5.890295 |
| H | -32.526632 | 70.226790 | -5.319417 |
| C | -33.040212 | 67.926342 | -7.461404 |
| N | -32.252683 | 68.879398 | -6.968408 |
| H | -32.824426 | 67.302931 | -8.313935 |
| C | -29.742971 | 72.440069 | -4.574653 |
| H | -30.167963 | 73.419237 | -4.777899 |
| H | -30.174987 | 72.030169 | -3.659687 |
| H | -28.664378 | 72.503401 | -4.433060 |
| C | -30.059509 | 71.489900 | -5.728468 |
| O | -30.883012 | 71.803752 | -6.609667 |
| O | -29.480014 | 70.335450 | -5.751489 |
| C | -28.567379 | 65.394261 | -8.102668 |
| H | -28.881700 | 64.356557 | -8.009607 |
| H | -28.348777 | 65.627623 | -9.143970 |
| H | -27.659051 | 65.555906 | -7.520890 |
| C | -29.623267 | 66.360194 | -7.592706 |
| O | -29.464664 | 67.597564 | -7.762865 |
| N | -30.675003 | 65.852984 | -6.960446 |
| H | -31.377808 | 66.478469 | -6.590000 |
| H | -30.906267 | 64.865173 | -6.906903 |
| C | -31.149851 | 72.623598 | -11.993618 |
| H | -31.614494 | 73.440892 | -11.444697 |
| H | -30.301119 | 73.025912 | -12.550307 |
| H | -31.881061 | 72.231935 | -12.703510 |
| C | -30.712661 | 71.562990 | -11.023787 |
| N | -30.077890 | 70.401123 | -11.427705 |
| H | -29.846416 | 70.156599 | -12.377312 |
| C | -30.807602 | 71.448272 | -9.662251 |
| H | -31.224482 | 72.128303 | -8.937933 |
| C | -29.819900 | 69.643397 | -10.341495 |
| N | -30.248483 | 70.250383 | -9.254798 |
| H | -29.332915 | 68.682653 | -10.369490 |
| Zn | -30.355418 | 69.491193 | -7.334519 |
| C | -33.623403 | 61.484578 | -5.654246 |
| H | -32.562865 | 61.258785 | -5.756568 |
| H | -33.847330 | 61.645174 | -4.596571 |
| H | -34.232229 | 60.652213 | -6.008897 |
| C | -33.976298 | 62.787246 | -6.424800 |
| O | -35.190263 | 62.936574 | -6.779225 |
| O | -33.045527 | 63.610723 | -6.609717 |
| C | -38.554910 | 61.299903 | -5.466858 |
| H | -37.657697 | 60.687497 | -5.356582 |
| H | -38.343641 | 62.272209 | -5.017527 |
| H | -39.368858 | 60.829829 | -4.916464 |
| C | -38.938258 | 61.440105 | -6.884146 |
| N | -38.128565 | 61.975933 | -7.857596 |
| H | -37.182731 | 62.335387 | -7.692053 |
| C | -40.097973 | 61.098034 | -7.509683 |
| H | -40.981821 | 60.641813 | -7.095610 |
| C | -38.803593 | 61.946141 | -9.022170 |
| N | -40.008467 | 61.417158 | -8.845367 |
| H | -38.408968 | 62.320002 | -9.951962 |
| C | -43.572723 | 57.880788 | -7.756585 |
| H | -43.130590 | 56.899329 | -7.608791 |
| H | -43.759030 | 58.365622 | -6.797390 |
| H | -44.529404 | 57.794321 | -8.273671 |

| | | | |
|---|---|---|---|
| C | -42.627914 | 58.750910 | -8.590853 |
| O | -41.555335 | 58.301579 | -9.013968 |
| O | -43.002581 | 59.973717 | -8.824793 |
| C | -42.933860 | 64.780006 | -11.501683 |
| H | -42.768484 | 65.830087 | -11.267158 |
| H | -42.641535 | 64.575824 | -12.531918 |
| H | -43.990170 | 64.533604 | -11.393459 |
| C | -42.143177 | 63.868160 | -10.587639 |
| O | -42.245814 | 62.616969 | -10.787626 |
| N | -41.387271 | 64.383813 | -9.639977 |
| H | -40.875694 | 63.739829 | -9.049029 |
| H | -41.220844 | 65.391239 | -9.409961 |
| C | -38.932353 | 57.518419 | -13.592652 |
| H | -38.779144 | 56.732176 | -12.855242 |
| H | -37.952248 | 57.875357 | -13.916282 |
| H | -39.444423 | 57.081442 | -14.452686 |
| C | -39.737348 | 58.617458 | -12.975988 |
| N | -40.107421 | 59.759527 | -13.664205 |
| H | -39.891365 | 59.964576 | -14.626570 |
| C | -40.256486 | 58.780456 | -11.721651 |
| H | -40.223706 | 58.127354 | -10.865291 |
| C | -40.812708 | 60.556338 | -12.834060 |
| N | -40.920042 | 59.989076 | -11.651305 |
| H | -41.224527 | 61.514503 | -13.105278 |
| Zn | -41.647771 | 60.861875 | -9.942943 |
| O | -35.931864 | 65.801205 | -7.175689 |
| H | -36.893332 | 65.905196 | -7.342271 |
| H | -35.694869 | 64.854911 | -7.055536 |

Model_4
110

| | | | |
|---|---|---|---|
| C | 5.136753 | -1.951802 | 2.072020 |
| H | 5.487952 | -2.292242 | 3.048165 |
| H | 4.732391 | -2.818065 | 1.546971 |
| H | 4.366083 | -1.193169 | 2.203603 |
| C | 6.333666 | -1.392450 | 1.290301 |
| O | 6.390661 | -0.171762 | 1.027490 |
| O | 7.208374 | -2.268315 | 0.981094 |
| C | 1.508863 | 1.795204 | 3.078185 |
| H | 1.873421 | 2.438163 | 3.881880 |
| H | 0.580672 | 1.328645 | 3.405149 |
| H | 2.249082 | 1.010919 | 2.907147 |
| C | 1.242917 | 2.577011 | 1.825994 |
| N | 2.184943 | 3.292937 | 1.109004 |
| H | 3.160401 | 3.473004 | 1.322519 |
| C | 1.584226 | 3.848400 | 0.042961 |
| N | 0.293128 | 3.545385 | 0.019098 |
| H | 2.135711 | 4.471330 | -0.642656 |
| C | 0.078979 | 2.747724 | 1.130766 |
| H | -0.896927 | 2.348443 | 1.339998 |
| C | -4.085754 | 1.861616 | 1.266699 |
| H | -3.656004 | 2.219924 | 2.205367 |
| H | -3.647259 | 0.886401 | 1.054359 |
| H | -5.165046 | 1.780231 | 1.376244 |
| C | -3.721404 | 2.832738 | 0.177274 |
| O | -2.497397 | 2.811021 | -0.167229 |
| O | -4.593195 | 3.589891 | -0.309465 |
| C | 1.396524 | 6.958717 | -2.952180 |
| H | 2.374454 | 7.220151 | -2.546848 |

| | | | |
|---|---|---|---|
| H | 0.907903 | 7.859592 | -3.331149 |
| H | 1.496695 | 6.251339 | -3.773653 |
| C | 0.520867 | 6.361600 | -1.891296 |
| O | -0.451177 | 5.633203 | -2.224665 |
| N | 0.797361 | 6.677907 | -0.633722 |
| H | 0.225609 | 6.265070 | 0.092589 |
| H | 1.766961 | 6.942412 | -0.401396 |
| C | -2.618775 | -0.325059 | -4.775590 |
| H | -1.832444 | -0.826895 | -5.342815 |
| H | -3.475790 | -0.161374 | -5.431846 |
| H | -2.929765 | -0.985685 | -3.967943 |
| C | -2.125431 | 0.975441 | -4.191476 |
| N | -1.662516 | 2.016924 | -4.977152 |
| H | -1.605220 | 2.015574 | -5.982735 |
| C | -1.304848 | 3.045136 | -4.173896 |
| N | -1.503502 | 2.734114 | -2.912813 |
| H | -0.921320 | 3.987750 | -4.529067 |
| C | -2.016183 | 1.449292 | -2.908791 |
| H | -2.273661 | 0.964123 | -1.982487 |
| Zn | -1.354745 | 4.079027 | -1.228457 |
| C | -3.734688 | 7.638804 | 0.967073 |
| H | -3.131019 | 7.896219 | 1.834465 |
| H | -3.945901 | 8.525083 | 0.365083 |
| H | -4.690881 | 7.222357 | 1.296209 |
| C | -3.031185 | 6.581758 | 0.134301 |
| O | -2.108552 | 5.903107 | 0.550009 |
| C | -3.527099 | 6.380053 | -1.291398 |
| H | -3.042215 | 7.138810 | -1.914177 |
| H | -4.607552 | 6.549965 | -1.329744 |
| O | -3.205048 | 5.108504 | -1.796540 |
| H | -3.839345 | 4.457283 | -1.329734 |
| C | 5.914397 | 7.247044 | -0.138492 |
| H | 5.731140 | 8.101358 | 0.514955 |
| H | 6.040472 | 7.625911 | -1.153850 |
| H | 6.811009 | 6.717298 | 0.179774 |
| C | 4.674654 | 6.318419 | -0.099633 |
| O | 4.804737 | 5.124677 | 0.286004 |
| O | 3.587193 | 6.850023 | -0.474127 |
| C | 10.117849 | 4.046728 | 1.834473 |
| H | 9.449334 | 3.777453 | 2.654275 |
| H | 9.694152 | 4.919197 | 1.332809 |
| H | 11.085962 | 4.324329 | 2.249882 |
| C | 10.286393 | 2.911839 | 0.889101 |
| N | 9.230996 | 2.315759 | 0.239586 |
| H | 8.229477 | 2.542162 | 0.376394 |
| C | 9.710956 | 1.333703 | -0.530542 |
| N | 11.032313 | 1.255101 | -0.416925 |
| H | 9.084411 | 0.686009 | -1.120368 |
| C | 11.401886 | 2.246371 | 0.468464 |
| H | 12.434081 | 2.424278 | 0.715020 |
| C | 15.991180 | 1.693388 | 0.149293 |
| H | 16.708084 | 0.885898 | -0.000959 |
| H | 15.814736 | 1.784558 | 1.222837 |
| H | 16.371384 | 2.630779 | -0.246938 |
| C | 14.661459 | 1.352535 | -0.545342 |
| O | 14.121149 | 2.192328 | -1.276443 |
| O | 14.131830 | 0.187548 | -0.313171 |
| C | 9.290532 | -3.277168 | -1.574132 |
| H | 8.345828 | -3.408088 | -1.049377 |
| H | 9.116687 | -3.037059 | -2.623635 |

| | | | |
|---|---|---|---|
| H | 9.877298 | -4.195982 | -1.526498 |
| C | 10.104618 | -2.156246 | -0.927479 |
| O | 11.182569 | -1.733503 | -1.468747 |
| N | 9.632870 | -1.683894 | 0.202271 |
| H | 10.153900 | -0.944106 | 0.653770 |
| H | 8.628750 | -1.896554 | 0.551553 |
| C | 13.033844 | 2.203750 | -6.425697 |
| H | 12.123219 | 2.524888 | -6.936194 |
| H | 13.688966 | 1.721753 | -7.154765 |
| H | 13.541166 | 3.090768 | -6.049722 |
| C | 12.726490 | 1.294563 | -5.278589 |
| N | 12.083261 | 0.077797 | -5.425363 |
| H | 11.766145 | -0.321974 | -6.293751 |
| C | 11.948156 | -0.490748 | -4.208534 |
| N | 12.472470 | 0.290368 | -3.287647 |
| H | 11.477222 | -1.441273 | -4.019108 |
| C | 12.961154 | 1.407159 | -3.937026 |
| H | 13.445103 | 2.191992 | -3.379927 |
| Zn | 12.383215 | -0.002110 | -1.253702 |
| O | 6.579380 | 2.814543 | 0.803842 |
| H | 5.944008 | 3.521209 | 0.573038 |
| H | 6.152368 | 1.941145 | 0.859872 |

Model_5$^{water}$

127

| | | | |
|---|---|---|---|
| C | 4.750915 | -2.222977 | 1.760012 |
| H | 5.009847 | -2.422355 | 2.803894 |
| H | 5.028754 | -3.095737 | 1.166428 |
| H | 3.678366 | -2.046353 | 1.697307 |
| C | 5.525262 | -0.981464 | 1.297225 |
| O | 4.922976 | 0.115002 | 1.323280 |
| O | 6.736356 | -1.148210 | 0.950210 |
| C | 1.641008 | 1.947937 | 2.709989 |
| H | 2.014657 | 2.644655 | 3.463022 |
| H | 0.814590 | 1.383168 | 3.139631 |
| H | 2.449282 | 1.258368 | 2.453090 |
| C | 1.154823 | 2.676858 | 1.492434 |
| N | 1.967346 | 3.396724 | 0.637501 |
| H | 2.966897 | 3.567658 | 0.721017 |
| C | 1.208604 | 3.900986 | -0.350763 |
| N | -0.063192 | 3.562537 | -0.194133 |
| H | 1.625453 | 4.503138 | -1.141361 |
| C | -0.100553 | 2.794386 | 0.956879 |
| H | -1.025058 | 2.365377 | 1.300899 |
| C | -3.871994 | 2.117009 | 1.726910 |
| H | -3.313037 | 2.800484 | 2.372648 |
| H | -3.329243 | 1.172969 | 1.685514 |
| H | -4.863541 | 1.970148 | 2.151845 |
| C | -3.936564 | 2.739798 | 0.375498 |
| O | -2.856676 | 2.641347 | -0.293692 |
| O | -4.967259 | 3.340650 | -0.000183 |
| C | 0.556157 | 7.043643 | -2.318025 |
| H | 1.498550 | 7.449671 | -1.952131 |
| H | 0.020839 | 7.798989 | -2.896481 |
| H | 0.744592 | 6.201933 | -2.986795 |
| C | -0.330460 | 6.573277 | -1.228593 |
| O | -1.412040 | 6.027633 | -1.567369 |
| N | 0.043682 | 6.739219 | 0.027502 |

| | | | |
|---|---|---|---|
| H | -0.521112 | 6.313843 | 0.752564 |
| H | 1.004850 | 7.048111 | 0.232952 |
| C | -3.102002 | -0.210964 | -4.444894 |
| H | -2.736707 | -0.440348 | -5.448216 |
| H | -4.184800 | -0.351566 | -4.426987 |
| H | -2.656694 | -0.923095 | -3.751980 |
| C | -2.732224 | 1.179719 | -4.026234 |
| N | -3.144225 | 2.309730 | -4.711691 |
| H | -3.696674 | 2.320425 | -5.553667 |
| C | -2.704456 | 3.401890 | -4.048792 |
| N | -2.021643 | 3.046303 | -2.983378 |
| H | -2.919295 | 4.415144 | -4.345364 |
| C | -2.031202 | 1.664994 | -2.958066 |
| H | -1.568738 | 1.120543 | -2.151959 |
| Zn | -1.890026 | 4.115965 | -1.162711 |
| C | -3.693992 | 7.714960 | 1.287991 |
| H | -2.965283 | 8.452955 | 0.938696 |
| H | -4.693859 | 8.109409 | 1.104533 |
| H | -3.536925 | 7.546329 | 2.351713 |
| C | -3.450721 | 6.423239 | 0.526979 |
| O | -2.687855 | 5.565678 | 0.935956 |
| C | -4.170560 | 6.271924 | -0.803449 |
| H | -3.874168 | 7.114906 | -1.434308 |
| H | -5.246118 | 6.362187 | -0.609478 |
| O | -3.888731 | 5.073569 | -1.476771 |
| H | -4.439661 | 4.338470 | -1.033076 |
| C | 6.586010 | 6.982962 | -0.323983 |
| H | 6.986125 | 7.372225 | 0.615315 |
| H | 5.909198 | 7.720778 | -0.757373 |
| H | 7.424000 | 6.794968 | -0.993699 |
| C | 5.865088 | 5.649777 | -0.026296 |
| O | 6.596449 | 4.657256 | 0.143161 |
| O | 4.588825 | 5.643790 | 0.049957 |
| C | 9.968037 | 3.533050 | 1.738997 |
| H | 9.594751 | 3.261733 | 2.728627 |
| H | 9.206015 | 4.155240 | 1.263610 |
| H | 10.883616 | 4.113262 | 1.854065 |
| C | 10.242008 | 2.323962 | 0.923745 |
| N | 9.256949 | 1.453821 | 0.530075 |
| H | 8.236722 | 1.579654 | 0.777481 |
| C | 9.817750 | 0.488599 | -0.199185 |
| N | 11.135279 | 0.677741 | -0.297818 |
| H | 9.253876 | -0.326869 | -0.619776 |
| C | 11.406303 | 1.835228 | 0.406081 |
| H | 12.394790 | 2.258207 | 0.446172 |
| C | 15.805009 | 1.545002 | 0.422996 |
| H | 16.624406 | 0.860819 | 0.196719 |
| H | 15.383265 | 1.254321 | 1.388863 |
| H | 16.170429 | 2.567741 | 0.485561 |
| C | 14.715652 | 1.424337 | -0.613120 |
| O | 14.160725 | 2.411122 | -1.086616 |
| O | 14.400196 | 0.191230 | -0.884787 |
| C | 10.094006 | -3.799996 | -1.704996 |
| H | 9.268788 | -4.145854 | -1.083753 |
| H | 9.718025 | -3.450492 | -2.665992 |
| H | 10.784221 | -4.625226 | -1.893622 |
| C | 10.859825 | -2.700361 | -1.051396 |
| O | 11.669686 | -2.046983 | -1.758271 |
| N | 10.656169 | -2.497797 | 0.237369 |
| H | 11.194078 | -1.788267 | 0.715297 |

| | | | |
|---|---|---|---|
| H | 9.822404 | -2.891721 | 0.694293 |
| C | 13.457008 | 2.389014 | -6.213023 |
| H | 12.610357 | 2.719032 | -6.819591 |
| H | 14.229181 | 2.000654 | -6.881411 |
| H | 13.862064 | 3.259649 | -5.699489 |
| C | 13.046943 | 1.376720 | -5.200024 |
| N | 12.499051 | 0.147680 | -5.520046 |
| H | 12.317571 | -0.195376 | -6.449216 |
| C | 12.248998 | -0.522244 | -4.373578 |
| N | 12.604913 | 0.209327 | -3.341208 |
| H | 11.829659 | -1.512899 | -4.317931 |
| C | 13.104189 | 1.394332 | -3.836749 |
| H | 13.469079 | 2.157902 | -3.167978 |
| C | 15.250004 | -2.425006 | 2.018007 |
| H | 15.815982 | -1.511614 | 2.224966 |
| H | 14.500891 | -2.555685 | 2.796503 |
| H | 15.953615 | -3.259666 | 2.017805 |
| C | 14.578842 | -2.259631 | 0.677212 |
| O | 13.433406 | -1.867882 | 0.579523 |
| C | 15.458997 | -2.602873 | -0.512760 |
| H | 16.438349 | -2.136818 | -0.333512 |
| H | 15.616421 | -3.686268 | -0.483412 |
| O | 14.939733 | -2.279983 | -1.766275 |
| H | 14.911465 | -1.307592 | -1.805758 |
| Zn | 12.545823 | -0.301831 | -1.336397 |
| O | 6.810104 | 2.148961 | 1.333947 |
| H | 6.528540 | 2.973083 | 0.884118 |
| H | 6.103121 | 1.465877 | 1.257805 |
| O | 2.768701 | 7.470945 | 0.126949 |
| H | 3.526653 | 6.786421 | 0.069894 |
| H | 3.115785 | 8.187262 | 0.669699 |
| O | 8.114094 | -3.335877 | 1.062694 |
| H | 7.867091 | -3.785838 | 1.877594 |
| H | 7.526670 | -2.500070 | 1.018991 |

Model_5

121

| | | | |
|---|---|---|---|
| C | 4.750993 | -2.222996 | 1.760011 |
| H | 4.815270 | -1.961241 | 2.819081 |
| H | 5.107254 | -3.243141 | 1.623807 |
| H | 3.707983 | -2.134123 | 1.453874 |
| C | 5.625969 | -1.221688 | 0.958940 |
| O | 5.130854 | -0.072075 | 0.775148 |
| O | 6.762329 | -1.616918 | 0.579306 |
| C | 1.641020 | 1.947993 | 2.710013 |
| H | 2.603448 | 2.355711 | 3.025708 |
| H | 0.884247 | 2.247060 | 3.434161 |
| H | 1.723722 | 0.859164 | 2.721853 |
| C | 1.255290 | 2.444523 | 1.368426 |
| N | 1.979327 | 2.224199 | 0.217309 |
| H | 2.870696 | 1.741012 | 0.164680 |
| C | 1.316546 | 2.788867 | -0.811849 |
| N | 0.202204 | 3.359715 | -0.385708 |
| H | 1.678244 | 2.784179 | -1.826534 |
| C | 0.159584 | 3.152594 | 0.975472 |
| H | -0.665191 | 3.505506 | 1.570497 |
| C | -3.871947 | 2.117082 | 1.726922 |
| H | -3.328153 | 2.572230 | 2.558530 |

| | | | |
|---|---|---|---|
| H | -3.437157 | 1.134788 | 1.546168 |
| H | -4.923704 | 2.033822 | 1.993074 |
| C | -3.683824 | 2.991493 | 0.513546 |
| O | -2.537099 | 2.914026 | -0.028459 |
| O | -4.611567 | 3.740651 | 0.121847 |
| C | 0.726977 | 7.210948 | -3.288936 |
| H | 1.745979 | 7.591138 | -3.349546 |
| H | 0.025093 | 8.042422 | -3.221252 |
| H | 0.490255 | 6.642814 | -4.189123 |
| C | 0.516701 | 6.321319 | -2.099984 |
| O | -0.653587 | 5.864235 | -1.953177 |
| N | 1.516248 | 6.069716 | -1.278263 |
| H | 1.324997 | 5.463529 | -0.491320 |
| H | 2.505526 | 6.387734 | -1.360548 |
| C | -3.101998 | -0.210987 | -4.444985 |
| H | -2.518876 | -0.575127 | -5.293464 |
| H | -4.156784 | -0.195987 | -4.727319 |
| H | -2.981349 | -0.916512 | -3.624450 |
| C | -2.639145 | 1.144810 | -4.002317 |
| N | -2.666859 | 2.254104 | -4.828368 |
| H | -2.980933 | 2.268819 | -5.785254 |
| C | -2.218457 | 3.323209 | -4.132197 |
| N | -1.895586 | 2.969159 | -2.909510 |
| H | -2.159161 | 4.322724 | -4.530823 |
| C | -2.154182 | 1.616655 | -2.812282 |
| H | -1.994284 | 1.090155 | -1.886027 |
| Zn | -1.548531 | 4.208590 | -1.214683 |
| C | -3.693990 | 7.714975 | 1.287997 |
| H | -3.045974 | 7.916597 | 2.138105 |
| H | -3.869014 | 8.625904 | 0.711702 |
| H | -4.662079 | 7.351879 | 1.644330 |
| C | -3.086624 | 6.633606 | 0.412479 |
| O | -2.211127 | 5.876410 | 0.785223 |
| C | -3.639464 | 6.514537 | -1.001833 |
| H | -3.124729 | 7.255506 | -1.620250 |
| H | -4.707921 | 6.752019 | -0.996701 |
| O | -3.419022 | 5.238860 | -1.555155 |
| H | -3.994274 | 4.591744 | -1.003580 |
| C | 6.585943 | 6.982989 | -0.324008 |
| H | 6.529234 | 7.954888 | -0.811986 |
| H | 7.461467 | 6.428720 | -0.664428 |
| H | 6.674315 | 7.126021 | 0.755662 |
| C | 5.301703 | 6.147672 | -0.600704 |
| O | 5.357729 | 4.920789 | -0.333082 |
| O | 4.292356 | 6.769238 | -1.050426 |
| C | 9.967994 | 3.532988 | 1.738980 |
| H | 9.279697 | 3.152511 | 2.496533 |
| H | 9.554081 | 4.469827 | 1.359922 |
| H | 10.924704 | 3.745010 | 2.215060 |
| C | 10.169309 | 2.551334 | 0.650210 |
| N | 9.183862 | 2.069781 | -0.177165 |
| H | 8.145676 | 2.247333 | -0.130075 |
| C | 9.749221 | 1.210407 | -1.032712 |
| N | 11.054873 | 1.103548 | -0.800266 |
| H | 9.192307 | 0.672494 | -1.782383 |
| C | 11.323660 | 1.947969 | 0.251844 |
| H | 12.319553 | 2.087258 | 0.632185 |
| C | 15.804987 | 1.544998 | 0.422962 |
| H | 16.695804 | 1.181525 | -0.095952 |
| H | 15.758275 | 1.061379 | 1.398571 |

| | | | |
|---|---|---|---|
| H | 15.880274 | 2.624583 | 0.529408 |
| C | 14.585677 | 1.197629 | -0.407409 |
| O | 14.062021 | 2.030724 | -1.146001 |
| O | 14.135717 | -0.024001 | -0.292412 |
| C | 10.094059 | -3.799938 | -1.705015 |
| H | 9.124021 | -4.177315 | -1.386100 |
| H | 10.130533 | -3.736191 | -2.792346 |
| H | 10.884261 | -4.476754 | -1.378014 |
| C | 10.398494 | -2.443069 | -1.134300 |
| O | 11.488127 | -1.911290 | -1.474708 |
| N | 9.539380 | -1.875565 | -0.303361 |
| H | 9.764236 | -0.968206 | 0.080946 |
| H | 8.588408 | -2.193756 | -0.095616 |
| C | 13.456984 | 2.388959 | -6.212933 |
| H | 12.726324 | 2.300124 | -7.019941 |
| H | 14.458241 | 2.369779 | -6.649019 |
| H | 13.316044 | 3.356990 | -5.734944 |
| C | 13.283218 | 1.315058 | -5.193715 |
| N | 13.404311 | -0.036228 | -5.465963 |
| H | 13.630132 | -0.441705 | -6.360042 |
| C | 13.193514 | -0.727481 | -4.325918 |
| N | 12.944657 | 0.105729 | -3.338623 |
| H | 13.233809 | -1.801660 | -4.246441 |
| C | 12.997915 | 1.380355 | -3.861931 |
| H | 12.858350 | 2.243146 | -3.233122 |
| C | 15.249976 | -2.425009 | 2.017991 |
| H | 15.051316 | -1.379191 | 2.269616 |
| H | 14.556139 | -3.059745 | 2.565744 |
| H | 16.281139 | -2.650430 | 2.299925 |
| C | 15.033282 | -2.578366 | 0.531397 |
| O | 14.000010 | -2.994219 | 0.056502 |
| C | 16.208432 | -2.149088 | -0.336436 |
| H | 16.692674 | -1.291858 | 0.147222 |
| H | 16.931412 | -2.970531 | -0.348113 |
| O | 15.856202 | -1.871711 | -1.665273 |
| H | 15.136987 | -1.223154 | -1.607506 |
| Zn | 12.483470 | -0.184452 | -1.371952 |
| O | 6.512669 | 2.260467 | -0.188328 |
| H | 6.005499 | 3.097618 | -0.160568 |
| H | 6.002497 | 1.495406 | 0.168024 |

## References

1. Harmer, N.J. The structure of sedoheptulose-7-phosphate isomerase from Burkholderia pseudomallei reveals a zinc binding site at the heart of the active site. *J Mol Biol* **400**, 379-92 (2010).
2. Jacobs, D.J., Rader, A.J., Kuhn, L.A. & Thorpe, M.F. Protein flexibility predictions using graph theory. *Proteins* **44**, 150-65 (2001).
